# MicroRNA and Alzheimer’s disease: Diagnostic biomarkers and potential therapeutic targets

**DOI:** 10.4103/NRR.NRR-D-25-00002

**Published:** 2025-07-05

**Authors:** Yiwen Huang, Yimin Chen, Zhengyang He, Wenfeng Lu, Hejin Lai, Yu Wang, Jie Wang

**Affiliations:** 1 Department of Chinese Medicine & Integrative Medicine, Shanghai Geriatric Medical Center, Zhongshan Hospital, Fudan University, Shanghai, China; 2 Department of Chinese Medicine & Integrative Medicine, Zhongshan Hospital, Fudan University, Shanghai, China; 3 Endocrinology Department of Shanghai Municipal Hospital of Traditional Chinese Medicine, Shanghai University of Traditional Chinese Medicine, Shanghai, China; 4 Department of College of Traditional Chinese Medicine, Tianjin University of Traditional Chinese Medicine, Tianjin, China; 5 CAS Key Laboratory of Nutrition, Metabolism and Food Safety, Shanghai Institute of Nutrition and Health, University of Chinese Academy of Sciences, Chinese Academy of Sciences, Shanghai, China; 6 Department of Neurology, Shuguang Hospital Affiliated to Shanghai University of Traditional Chinese Medicine, Shanghai, China

**Keywords:** Alzheimer’s disease, amyloid-β, diagnostic biomarker, glial cells, microRNA, neuroinflammatory, neuronal death, synapses, tau protein, therapeutic targets

## Abstract

MicroRNAs (miRNAs), small non-coding RNAs ranging from 19 to 25 nucleotides in length, are key regulators of gene expression that function primarily by inhibiting the translation of target mRNAs. Recent studies have suggested that miRNAs play important roles in regulating key aspects in the pathology of Alzheimer’s disease, including the modulation and accumulation of amyloid-beta and tau proteins. Moreover, miRNAs have been implicated in the regulation of neuroinflammation through various inflammatory pathways, notably the nuclear factor kappa B signaling cascade. Additional emerging evidence has shown that miRNAs regulate synaptic growth and maturation, and they perform promising roles in regulating neuronal death and development. miRNAs also offer a novel avenue for direct reprogramming of neurons, representing a promising strategy for Alzheimer’s disease treatment. The regulation of miRNA biogenesis and the post-transcriptional modifications of miRNAs are critical factors in Alzheimer’s disease pathology, influencing miRNA activity and disease progression. In this review, we comprehensively explore the role of different miRNAs in regulating various pathological processes associated with Alzheimer’s disease, focusing primarily on four representative miRNAs: miR-9, miR-29, miR-126, and miR-146a for further exploration. We also discuss the influence of miRNA biogenesis on Alzheimer’s disease, emphasizing how dysregulation of miRNA processing may contribute to the disease. Additionally, we highlight the potential of miRNAs as both diagnostic biomarkers and therapeutic targets in Alzheimer’s disease, along with promising vector delivery strategies aimed at improving clinical outcomes. Finally, we discuss the challenges and limitations associated with the use of miRNAs in the diagnosis and treatment of Alzheimer’s disease. By reviewing the current clinical applications of miRNAs as biomarkers and therapeutic agents, we aim to provide insights that will inform future research and development in this promising field.

## Introduction

Alzheimer’s disease (AD) is a progressively worsening neurodegenerative disorder that has become one of the most destructive and burdensome diseases worldwide. As a major type of dementia, AD requires ongoing and expensive treatment, with global cost estimates expected to increase several times by 2050 (Chen et al., 2024c). Patients with AD experience a range of cognitive-behavioral impairments, including deterioration in expression, decreased comprehension, and memory loss, all of which ultimately lead to an undignified death. The pathogenesis of AD primarily involves the amyloid cascade, intracellular neurofibrillary tangles of hyperphosphorylated tau, neuroinflammation of the two main glial cell populations (microglia and astrocytes), and synaptic dysfunction, with neuronal death ultimately occurring due to a complex interplay of factors (Rodriguez-Jimenez et al., 2023; Zhang et al., 2023; Wang et al., 2024a). However, in stark contrast to the pathologic mechanisms described above, the existing targeted drugs approved by the U.S. Food and Drug Administration (FDA) are limited to some “symptomatic” medications, including amyloid-beta (Aβ) inhibitors such as aducanumab and lecanemab (Harris, 2023), acetylcholinesterase inhibitors such as donepezil, rivastigmine, and galantamine, and N-methyl-D-aspartate antagonists like memantine (Larkin, 2022; Peng et al., 2023; Singh et al., 2024; Zheng and Wang, 2025). These drugs can only improve the cognitive and behavioral symptoms but cannot alter the underlying course of the disease (Zhang et al., 2024a). **Additional Table 1** lists all FDA-approved medications, as well as others in clinical phase III trials (completed), for the treatment of AD (Doody et al., 2014; Syed, 2020). Thus, a more comprehensive and microscopic perspective is needed to decipher this complex disease. In contrast to the unidirectional linear pathogenicity cascade that has been previously highlighted in the context of AD, newer perspectives emphasize the importance of intertwining complex cellular pathways, gene networks, and feed-forward regulatory loops that may differentially affect various pathogenicity endophenotypes and cellular phases of the disease (Aljassabi et al., 2024; Cummings et al., 2024; Zheng and Wang, 2025).

**Additional Table 1:** Drugs approved by the FDA and representative in the late clinical stage

Drug	Status/phase	Mechanism	Reference
Aducanumab	FDA approved	Aβ clearance	Sevigny et al., 2016
Lecanemab	FDA approved	Aβ clearance	Harris, 2023
Semagacestat	Phase III completed	Aβ clearance	Doody et al., 2013
ALZT-OP1	Phase III completed	Aβ clearance	NCT02547818
Solanezumab	Phase III completed	Aβ clearance	Doody et al., 2014
TRx0237	Phase III completed	Tau pathology	NCT01689233; NCT01689246
Sodium oligomannate	CFDA approved	Neuroinflammation triggered by intestinal flora dysbiosis and Aβ clearance	Syed, 2020
NE3107	Phase III completed	Neuroinflammation	NCT04669028
Donepezil	FDA approved	AChEI	Larkin, 2022
Rivastigmine	FDA approved	AChEI	Rösler et al., 1999
Galantamine	FDA approved	AChEI	Wilcock et al., 2000
Memantine	FDA approved	NMDA antagonist	Rodda and Carter, 2012
Nilvadipine	Phase III completed	Calcium channel blocker	NCT02017340

AChEIs: Acetylcholinesterase inhibitors; Aβ: amyloid-β; CFDA: China Food and Drug Administration; FDA: Food and Drug Administration; NCT: National Clinical Trial; NMDA: N-methyl D-aspartate antagonist.

One of the crucial regulators of gene expression is microRNAs (miRNAs) (Fu et al., 2023; Hwang et al., 2023). miRNAs are small RNA fragments, typically ranging from 19 to 25 nucleotides in length. A single miRNA can target hundreds of mRNAs and affect the expression of many genes, while one gene can be regulated by multiple miRNAs through interactions among different miRNAs (Shao et al., 2019). miRNAs serve as critical regulators in the progression and development of numerous diseases by modulating gene expression at the post-transcriptional level. Their roles have been extensively studied in conditions such as cancer and cardiovascular diseases, and miRNAs are being increasingly recognized for their therapeutic potential in neurodegenerative disorders. The influence of miRNAs on critical biological pathways makes them promising targets for intervention as well as valuable diagnostic and prognostic biomarkers across a broad spectrum of diseases. For instance, miR-29b has been identified as a key regulator in AD pathology due to its ability to reduce Aβ aggregation by targeting presenilin1 (PSEN1) (Wuli et al., 2022). miR-9 increases tau phosphorylation by decreasing the expression of ubiquitin conjugation E4B (UBE4B), and miR-124 blocks microglial pro-inflammatory responses by inhibiting Toll-like receptor (TLR) 4 and NOD-like receptor family pyrin domain-containing 3 (NLRP3) activity (Subramanian et al., 2021; Yang et al., 2022). miR-146a-5p causes synaptic alterations by downregulating synaptotagmin1 (*Syt1*) mRNA and neuroligin1 (*Nlg1*) mRNA, leading to decreased spontaneous miniature synaptic currents in receiving neurons (Prada et al., 2018).

In the present review, we show how miRNAs significantly influence AD pathogenesis by modulating key pathological processes, including Aβ aggregation, tau hyperphosphorylation, neuroinflammation, synaptic dysfunction, and neuron dysfunction. We have systematically examined the regulatory roles of miRNAs, offering insights into their potential as therapeutic targets. miR-132 mitigates Aβ accumulation by suppressing sirtuin 1 and inositol 1,4,5-trisphosphate 3-kinase B (ITPKB), thereby modulating neuroprotective signaling pathways relevant to AD pathology (Hernandez-Rapp et al., 2016; Salta et al., 2016; Zeng et al., 2022a). miR-4422-5p, miR-195, and miR-342-5p suppress β-site amyloid precursor protein (APP)-cleaving enzyme-1 (BACE1) expression, mitigating Aβ accumulation, a critical factor in AD pathogenesis (Zhu et al., 2012; Hajjari et al., 2021; Dong et al., 2022). Other miRNAs have been shown to regulate Aβ accumulation through distinct molecular targets. miR-137-5p attenuates Aβ deposition in the hippocampal and cortical regions by inhibiting ubiquitin-specific peptidase 30, while miR-128 modulates glycogen synthase kinase-3β (GSK-3β), APP binding protein 2, and mechanistic target of rapamycin (mTOR) to suppress amyloid production (Jiang et al., 2023; Li et al., 2023a). miR-143-3p and miR-539-5p regulate death-associated protein kinase 1 (DAPK1) and the phosphatidylinositol 3-kinase (PI3K)/protein kinase B (Akt)/GSK-3β pathway, respectively, to inhibit aberrant amyloidogenic processing of APP (Jiang et al., 2020b; Wang et al., 2022). This regulation ultimately reduces Aβ protein production and the associated memory impairment. Conversely, miR-30a-5p and miR-138 contribute to the generation of Aβ by directly regulating a disintegrin and metalloproteinase domain-containing protein 10 (ADAM10) (Lu et al., 2019; Sun et al., 2022). Meanwhile, both miR-429-3p and miR-342-3p act as bad actors, regulating mitogen-activated protein kinase phosphatase 1 and c-Jun N-terminal kinase (JNK), respectively, enhancing amyloidogenic APP processing and Aβ accumulation (Fu et al., 2019; Luo et al., 2024). Through an in-depth analysis of the relationships of miRNAs with tau proteins, studies have identified numerous miRNAs that are involved in tau regulation. Understanding these regulatory interactions can also provide valuable insights into potential therapeutic strategies. miR-23b may attenuate tau phosphorylation and toxicity by regulating GSK-3β and Akt expression, highlighting the role of the Akt/GSK-3β pathway in tau pathology (Pan et al., 2021; Jiang et al., 2022). miR-101b, miR-143-3p, miR-369, and miR-200c regulate AMP-activated kinase (AMPK), DAPK1, Fyn and serine/threonine protein kinase 2, and 14-3-3 protein gamma isoform (14-3-3γ), respectively, collectively mitigating tau hyperphosphorylation (Liu et al., 2017a; Yao et al., 2019b; Park et al., 2022; Wang et al., 2022). In contrast, miR-140 suppresses phosphatase and tensin homolog (PTEN) induced putative kinase 1, with miR-125b downregulating dual-specific phosphatase 6 and protein phosphatase 1 catalytic subunit alpha isoform, promoting tau hyperphosphorylation and aggregation, and further driving neurodegeneration (Banzhaf-Strathmann et al., 2014; Liang et al., 2021a). Aberrant protein accumulation is known to induce neuroinflammation, which is exacerbated by miR-98-5p regulating α7 nicotinic acetylcholine receptor and nuclear factor erythroid 2-related factor 2 through the nuclear factor kappa B (NF-κB) signaling pathway. In contrast, upregulation of miR-485-3p, miR-224-5p, miR-216a-5p, miR-331-3p, and miR-212-3p *in vivo* has been shown to attenuate neuroinflammation by reducing the secretion of pro-inflammatory cytokines (Koh et al., 2021; Liu and Lei, 2021; Shao, 2021; Song et al., 2021; Nong et al., 2022; Sun et al., 2023). Importantly, researchers have identified and summarized a group of miRNAs that play crucial roles in regulating synaptic function. Among them, miR-204-3p inhibits nicotinamide adenine dinucleotide phosphate (NADPH) oxidase 4 (Nox4), effectively rescuing synaptic dysfunction and memory impairment in the hippocampus of APPswe/presenilin protein (PS) 1dE9 mice (Tao et al., 2021). Similarly, miR-132 regulates multiple targets, including dedicator of cytokinesis (Dock)1, ephrin receptor B3, B-cell translocation gene anti-proliferation factor 2, calcium/calmodulin-dependent protein kinase 1, and Ras-related C3 botulinum toxin substrate 1 (Rac1), thereby promoting adult hippocampal neurogenesis and alleviating the memory deficits associated with AD (Walgrave et al., 2021). Additionally, miR-135a-5p improves synaptic integrity and cognitive function by modulating the Rho-associated coiled-coil containing protein kinase (ROCK)2/Adducin 1 signaling pathway (Zheng et al., 2021a). Another group of miRNAs containing miR-455-5p, miR-34c, and miR-431 has been identified as mediators of synaptic deficits and loss by regulating key synaptic regulatory proteins, including α-amino-3-hydroxy-5-methyl-4-isoxazole propionic acid (AMPA) receptor, a crucial component of excitatory synaptic transmission, Syt1, and Kremen1 (Krm1) (Ross et al., 2018; Shi et al., 2020; Xiao et al., 2021). These miRNAs play detrimental roles in synaptic integrity, further contributing to cognitive decline and neurodegeneration. Despite the inevitable neuron death in AD, numerous miRNAs continue to regulate this process, offering potential therapeutic targets. miR-107 (fibroblast growth factor 7), miR-130a-3p (DAPK1), and miR-21 (programmed cell death protein 4) mitigate Aβ-induced inflammation and apoptosis in SH-SY5Y cells, highlighting their neuroprotective roles (Feng et al., 2018; Chen et al., 2020c; Wang et al., 2021). The PI3K/Akt signaling pathway, a critical regulator of cell survival and apoptosis, is influenced by miR-212 and miR-4763-3p (Wang and Chang, 2020; Qi et al., 2024a); elevated levels of both miRNAs have been shown to contribute to decreased neuronal apoptosis. Similarly, miR-142-5p (protein tyrosine phosphatase non-receptor type (PTPN)1/Akt), miR-188 (nitric oxide synthase 1), miR-132-3p (heterogeneous nuclear ribonucleoprotein U), and miR-326 (Vav guanine nucleotide exchange factor 1/JNK) inhibit neuronal apoptosis in AD models, underscoring their therapeutic potential (Chen et al., 2020b; He et al., 2020; Qu et al., 2021; Liang et al., 2023a). In contrast to neuroprotective miRNAs, certain miRNAs such as miR-6076 (B-cell lymphoma 6), miR-125b (suppressed sphingosine kinase 1), miR-590-3p (AMPK), and miR-10b-5p (homeobox D10) promote neuronal apoptosis, highlighting their role in neurodegeneration (Jin et al., 2018; Cao et al., 2021a; Ruan et al., 2021; Lin et al., 2023b). However, viewed from another angle, miR-103 (prostaglandin-endoperoxide synthase 2) and miR-26a (PTEN) have also been shown to promote neurite growth, supporting neuronal plasticity and regeneration (Li and Sun, 2013; Yang et al., 2018a). To gain deeper insights into the complex regulatory mechanisms of miRNAs in AD, we have selected four representative miRNAs from a large pool of identified candidates, namely miR-9, miR-29, miR-126, and miR-146a. By focusing on these specific miRNAs as well as their structure and function, we aim to clarify their molecular targets, signaling pathways, and potential therapeutic implications, thereby enhancing the existing understanding of miRNA-mediated regulation in AD progression.

## Search Strategy

We searched PubMed for all peer-reviewed articles published in English up to January 2025 using the following keywords: “Alzheimer disease,” “dementia,” “AD,” “small RNA,” “microRNA,” “miRNA,” “microRNA-9,” “microRNA-29,” “microRNA-124,” “microRNA-146a,” “miR-9,” “miR-29,” “miR-124,” “miR-146a,” and “neuron.” These searches were followed by further screening based on title and abstract content. Articles that focused solely on miRNAs or AD without exploring the relationships between the two were excluded.

## Research Progress of MicroRNAs

### Evolution and structure of miRNAs

The discovery of miRNAs by Victor Ambros in 1993 fundamentally transformed our understanding of gene regulation (Lee et al., 1993). Prior to this discovery, the role of noncoding RNAs was not well appreciated, and the focus of genetic research was primarily on protein-coding genes. The identification of the first miRNA provided crucial insights into how these small RNA molecules regulate gene expression at the posttranscriptional level, influencing various biological processes, such as development, differentiation, and disease progression. This breakthrough leads to a wealth of subsequent research that has revealed the complexity and significance of miRNAs in regulating cellular functions. Lsy-6 was the first miRNA identified as being involved in the development of the nervous system (Johnston and Hobert, 2003). This finding highlights a new avenue for the widespread influence of noncoding RNAs, such as miRNAs, on the formation and function of the nervous system. The miRNA pathway has been identified as a regulator of tau protein-induced neurodegeneration, providing new insights into the molecular mechanisms underlying neurodegenerative diseases (Bilen et al., 2006). This discovery also suggests a potential link between miRNAs and AD. As miRNAs can regulate gene expression at the posttranscriptional level, new avenues for therapeutic exploration in AD and related disorders have been identified. Since advancements in miRNA research related to the pathological mechanisms of AD, Vierbuchen et al. (2010) have led to groundbreaking discoveries in regenerative medicine. Notably, for the first time, researchers have successfully used identified factors to directly convert fibroblasts into functional neurons, a remarkable achievement with significant therapeutic implications for AD. This approach represents a promising avenue for developing treatments aimed at replacing damaged neurons in neurodegenerative diseases such as AD. Alarcón et al. (2015) were the first to demonstrate N^6^-methyladenosine (m^6^A) labeling of primary miRNAs (pri-miRNAs), critical modifications involved in their processing. This discovery marks a significant milestone in the understanding of miRNA modifications, shedding light on the role of m^6^A in regulating miRNA maturation and its broader implications in disease mechanisms. The elucidation of this modification opens new avenues for exploring how miRNA alterations contribute to various diseases, highlighting the importance of posttranscriptional modifications in the fine-tuning of gene expression and cellular function.

**Figure 1:**
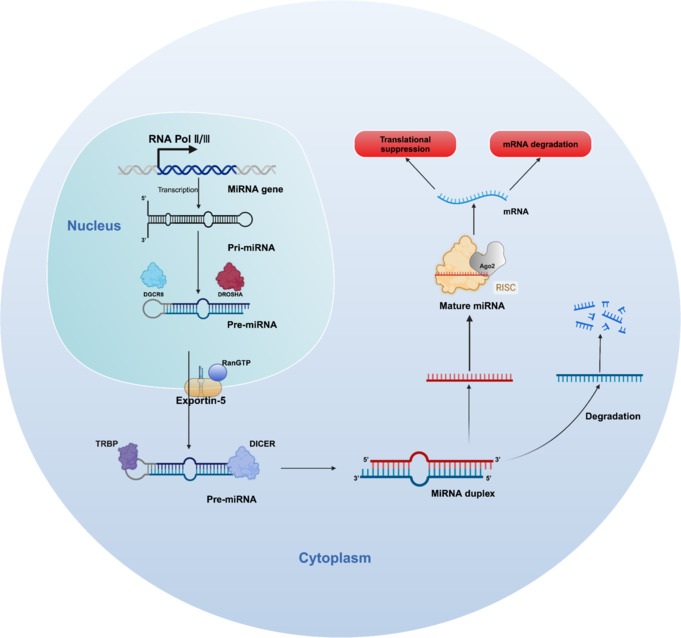
Processing and function of miRNAs. miRNA is first transcribed by RNA polymerase II or III in the nucleus and then transformed into Pri-miRNA, which mainly consists of a cap structure and a poly(A) tail. The Pri-miRNA is then recognized by DGCR8 and cleaved in conjunction with the Drosha protein to form a hairpin-shaped pre-miRNA. The exportin 5 protein subsequently transports the pre-miRNA into the cytoplasm in an exportin 5/RanGTP-dependent manner. In the cytoplasm, DICER and TRBP disassemble the pre-miRNA to form miRNA double strands, one of which is degraded, and the other binds to Ago family proteins to form the miRISC. Ultimately, miRISC targets the transcribed mRNA and inhibits its translation or direct degradation. Created with BioRender.com. Ago: Argonaute; DGCR8: DiGeorge syndrome critical region gene 8; DICER: Dicer enzyme; Drosha: Drosha enzyme; Pre-miRNA: precursor miRNA; Pri-miRNA: primary miRNA; RISC: RNA-induced silencing complex; TRBP: TAR RNA-binding protein.

miRNA biogenesis begins with RNA polymerase II-dependent (major) or RNA polymerase III-dependent transcription, which yields long primary transcripts containing typical hairpin structures (pri-miRNAs). Subsequently, DiGeorge syndrome critical region gene 8 (DGCR8) functions as a molecular anchor, recognizing pri-miRNAs at stem single-stranded RNA junctions and localizing the RNase III nucleic acid endonuclease Drosha at the correct catalytic site to cleave **~**11 bp from the junction, releasing a hairpin pre-miRNA (Kim et al., 2009). The pre-miRNA is then cleaved by one or more RNase III enzymes into mature miRNA, which is subsequently loaded onto the Argonaute protein to form the RNA-induced silencing complex (Amato et al., 2010) involved in post-transcriptional gene silencing (Kim et al., 2009; Yang and Lai, 2011). The processing and function of miRNAs are shown in **Figure 1**.

### Evolution, structure, and biological functions of miR-9

miR-9 is a class of miRNAs that is highly conserved in vertebrates and is transcribed from three separate genomes located on chromosomes 1q22 (MIR9-1), 5q14.3 (MIR9-2), and 15q26.1 (MIR9-3) (Radhakrishnan and Alwin Prem Anand, 2016; Roese-Koerner et al., 2016). Krichevsky et al. (2003) reported that miR-9 is dysregulated in progerin-1-deficient mice, which exhibit severe brain developmental defects, preliminarily revealing the role of miR-9 in neurodegenerative diseases. Researchers have reported that miR-9 plays a crucial role in regulating the equilibrium between the proliferation and differentiation of neural stem cells (Zhao et al., 2009). In parallel, it was also revealed that miR-9 governs both the proliferation and migration of neural progenitor cells derived from human embryonic stem cells (Delaloy et al., 2010). This dual regulatory function of miR-9 highlights its importance in neurogenesis, suggesting that it not only facilitates the expansion of neural progenitors but also coordinates their movement, which is essential for proper brain development. Similarly, Giusti et al. (2014) demonstrated that the role of miR-9 in downregulating the transcriptional repressor RE1-silencing transcription factor (REST) is vital for the proper growth of dendrites. The interaction between miR-9 and REST opens new avenues for understanding how miRNAs contribute to neuronal development and the pathophysiology of neurodevelopmental disorders. Abernathy et al. (2017) revealed that the interaction between miRNAs (miR-9) and transcription factors specific to distinct neuronal subtypes can drive the reprogramming of neural lineages, enabling the conversion of one type of neuron into another. This modular synergy facilitates the targeted generation of various human neuronal subtypes, offering a promising approach for regenerative medicine and disease modeling. Using a mouse model, Chen et al. (2021) demonstrated that the inhibition of miR-9-5p effectively halted the progression of AD. This protective effect was attributed to an increase in the autophagic clearance of Aβ. By promoting the degradation of Aβ plaques, miR-9-5p inhibition may help reduce neuronal damage and RelA, suggesting a potential therapeutic strategy for AD. In the following phase, Subramanian et al. (2021) pinpointed *UBE4B* mRNA as a direct target of miR-9. This discovery was significant, as UBE4B, together with STIP1 homology and U-Box containing protein 1 (STUB1), was found to play a key role in enhancing autophagic pathways that facilitate the degradation of the tau protein. Tau accumulation is another hallmark of AD, and its clearance is crucial for preventing neurodegeneration. By promoting the autophagic degradation of tau, miR-9 regulates UBE4B and STUB1.

In addition to its specific expression in the brain and peripheral nervous system and its ability to regulate many nerve cell functions, miR-9 also has important functions in other diseases, such as cancer. miR-9 has been reported to target the mRNA of suppressor of cytokine signaling 3 (SOCS3) in breast cancer, thereby increasing granulocytic myeloid-derived suppressor cell recruitment and leading to tumor proliferation (Gu et al., 2024). In patients with lung cancer, inhibition of TGFBR2 by miR-9-5p increases cancer cell growth and metastasis; conversely, in clear cell renal cell carcinoma, miR-9-5p prevents further tumor progression by inhibiting hyaluronan-mediated motility receptors, which promote the ability of cancer cells to proliferate and migrate (Li et al., 2017a; Niu et al., 2025). In neurodegenerative diseases such as Parkinson’s disease, miR-9-5p can reduce neuronal apoptosis by regulating scribble, a planar cell polarity protein, through the β-catenin signaling pathway to improve motor function in mice (Xiao et al., 2022). In brain-injured mice, hydrogen sulfide exerts neuroprotective effects, which reduce neuroinflammation through the miR-9-5p/C–X–C motif chemokine ligand (CXCL)11 axis (Zhang et al., 2024b).

### Evolution, structure, and biological functions of miR-29

In early investigations, Smirnova et al. (2005) reported that miR-29 is more abundantly expressed in astrocytes than in neurons. This differential expression pattern led to the hypothesis that miR-29 may play an essential role in regulating astrocyte differentiation, maturation, and ongoing functional maintenance within the central nervous system. Astrocytes, as key glial cells, are involved in supporting neuronal function, synaptic plasticity, and neuroprotection. The deletion of the miR-29a/b-1 cluster was found to result in increased expression of BACE1 (Hébert et al., 2008). These findings provide further evidence of the involvement of the miR-29 family in AD pathophysiology, highlighting its role in regulating critical enzymes involved in Aβ production. Research has shown that members of the miR-29 family, specifically miR-29a, miR-29b, and miR-29c, exhibit reduced expression levels in the gray matter of individuals diagnosed with AD (Wang et al., 2011). The diminished expression of these miRNAs in AD may contribute to the neurodegenerative processes observed in this disease, highlighting their potential as biomarkers or therapeutic targets. Ouyang et al. (2013) demonstrated that by suppressing the p53-upregulated modulator of apoptosis, miR-29a helps protect neurons from cell death. This discovery emphasizes the importance of astrocyte-derived miRNAs in regulating neuronal survival under pathological conditions. Jahangard et al. (2020) reported that exosomal miR-29 plays a crucial role in the regulation of apoptosis by targeting the 3′-untranslated region (UTR) of the Bcl-2-interacting mediator of cell death (*BIM*) mRNA. Understanding the molecular dynamics of the modulation of *BIM* translation by miR-29 offers promising therapeutic insights, particularly in neurodegenerative disorders characterized by dysregulated apoptosis. Recently, it was discovered that miR-29 influences microglial phagocytic activity through the modulation of mRNA expression. This discovery highlights the precise molecular targets of miR-29, which could provide novel insights into dysfunctional microglial activity (Scoyni et al., 2024).

The miR-29 family includes miR-29a, miR-29b-1, miR-29b-2, and miR-29c, of which miR-29b-1 and miR-29b-2 share the same mature sequence and are collectively called miR-29b. The miR-29 family, comprising miR-29a, miR-29b1 (both from chromosome 7q32.3), and miR-29b2, miR-29c (both from chromosome 1q32.2), shares a conserved seed sequence, AGCACC, across all four members (Smyth et al., 2022). This sequence plays a crucial role in their regulatory activities, influencing gene expression, apoptosis, and disease progression, such as heart disease and cancer. Despite their distinct chromosomal locations, the conserved seed region facilitates their functional interdependence. miR-29b-3p has been implicated in exacerbating cardiovascular disease by inhibiting cardiomyocyte proliferation and differentiation, a process mediated through the repression of Notch homolog (NOTCH)2, a critical regulator of cardiac development (Yang et al., 2020b). This mechanism underscores the potential of targeting miR-29b-3p in therapeutic strategies for cardiovascular conditions. Endothelial cells are fundamental to vascular integrity, as they regulate blood flow, maintain the blood‒brain barrier, and prevent abnormal clotting. Disruption of endothelial cell stability can significantly impair vascular function, leading to various cardiovascular conditions, including atherosclerosis. One mechanism through which endothelial dysfunction occurs is the activation of apoptotic pathways, particularly in response to inflammatory cytokines such as tumor necrosis factor-α (TNF-α). miR-29a has been identified as a key regulator in this process. Upon TNF-α stimulation, miR-29a mediates the apoptosis of endothelial cells, promoting their death (Cao et al., 2024). Evidence from *in vivo* experiments on neuroblastoma suggests that targeting B7-*H3* mRNA by miR-29 family members increases the infiltration of natural killer cells and CD8^+^ T cells and reduces tumor progression through the apoptotic pathway (Pathania et al., 2024). Ma et al. (2022) demonstrated that the inhibition of miR-29b increased Bcl-2 expression and inhibited apoptosis and oxidative damage in a model of ischemic stroke, and these findings suggest that miR-29b may act as a negative regulator of neuroprotection and that its inhibition may be a potential therapeutic strategy to mitigate the effects of ischemic stroke.

### Evolution, structure, and biological functions of miR-124

The existence and importance of miRNAs were completely known until 1993, when Victor Ambros discovered the first miRNA in the world, thus opening a new chapter in human research on such small RNAs (Lee et al., 1993). The discovery and study of miR-124 has also gradually unfolded in this broader context. Lagos-Quintana et al. (2002) discovered a new class of conserved miRNAs, named miR-124, by identifying mouse tissue-specific miRNAs. The following year, Krichevsky et al. (2003) used miRNA arrays to find a gradual increase in miR-124a signaling during embryonic development, followed by stable and high expression in adulthood, revealing a role for miR-124 in neuronal development and potential promise in neurological disorders. Shortly thereafter, miR-124 was recognized as a neuron-specific miRNA (Smirnova et al., 2005). Smith et al. (2011) reported reduced miR-124 expression in the brains of AD patients and thus affected the production of Aβ peptides, which contributes to the understanding of miR-124 and the importance of miRNAs in the pathogenesis of AD. In addition, Ponomarev et al. (2011) reported for the first time that miR-124 is expressed not only in neurons but also in microglia and that it is a key regulator of microglial quiescence. Exosomes can promote the transfer of miR-124a from neurons to astrocytes, a novel mechanism that is relevant for therapy, although astrocytes do not express miR-124 (Morel et al., 2013). Xue et al. (2013) demonstrated that miR-124 can reprogram fibroblasts into astrocytes, which has led to a new understanding of the therapeutic use of miR-124 in the treatment of neurological diseases such as AD. The discovery that the miR-124/PTPN1 signaling pathway is a key regulator of AD synaptic and memory impairment (Wang et al., 2018). Recently, Li et al. (2023b) demonstrated that combined exercise and cognitive training significantly enhances cognitive function in aged rats through a mechanism involving the targeted regulation of the long noncoding RNA (lncRNA) nuclear paraspeckle assembly transcript 1 (NEAT1)/miR-124-3p axis, suggesting a novel strategy for modulating cognitive decline through nonpharmacological interventions. Shortly thereafter, a study revealed that miR-124 in the exosomes of M2 microglia could reduce glutamate-induced neuronal death (Zhu et al., 2024). Évora et al. (2025) successfully developed the first tri-cell microfluidic coculture model of the central nervous system (CNS) by innovatively integrating neurons, microglia, and astrocytes and systematically validated the neuroprotective effects of miR-124-3p-enriched exosomes in AD via this model.

As initially discovered in mice, miR-124 is a highly conserved and tissue-specific miRNA predominantly found in the nervous system across various species (Lagos-Quintana et al., 2002; Sempere et al., 2004; Nelson et al., 2006). The three immature precursors of miR-124 are located on corresponding chromosomes (Lagos-Quintana et al., 2002). miR-124 regulates key biological processes, such as cellular proliferation, apoptosis, and migration, by targeting various downstream genes in several diseases. Metastasis in non-small cell lung cancer is potently inhibited by miR-124-3p, which acts by modulating both the intracellular PI3K/Akt signaling pathway and the extracellular conveyance of exosomes (Zhu et al., 2023). Similarly, a study revealed that miR-124 suppresses non-small cell lung carcinoma growth by targeting *Akt1* mRNA and *Akt2* mRNA and blocking the Akt pathway, and a miR-124 agomiR delivered systemically effectively suppresses tumorigenesis in K-ras LA1 transgenic mice (Jin et al., 2017). miR-124 plays an important role in hepatocellular carcinoma metastasis. The overexpression of miR-124 decreases chloride intracellular channel 1 expression in hepatocellular carcinoma cells and reduces hepatocellular carcinoma cell migration and invasion (Yue et al., 2019). Ning et al. (2014) reported that miR-124 is transcriptionally upregulated by hepatocyte nuclear factor 4α (HNF4α) and that increased HNF4α expression inhibits the metastasis of hepatocellular carcinoma cells both *in vitro* and *in vivo*. In Parkinson’s disease, miR-124 affects apoptosis and autophagy by regulating the BIM protein, subsequently reducing the loss of dopaminergic neurons in the central nervous system (Wang et al., 2016b). Glioma is one of the most malignant primary tumors of the brain and has rapid cell growth and immunosuppressive capabilities. miR-124 inhibits signal transducer and activator of transcription 3 (STAT3) signaling, a key regulatory molecule in microglia/macrophage-mediated immunosuppression, to enhance T-cell-mediated immune clearance in glioma (Wu et al., 2010; Wei et al., 2013). Additionally, Jiang et al. (2020a) showed that neuron-secreted miR-124-3p mitigated spinal cord trauma by inhibiting microglial activation via the PI3K/Akt/NF-κB signaling cascade.

### Evolution, structure, and biological functions of miR-146a

In the context of miR-146a, Li et al. (2011) first discovered that miRNA-146a regulates inflammation by targeting complement factor H (*CFH*) mRNA, interleukin-1 receptor-associated kinase-1 (IRAK-1) mRNA, and *tetraspanin-12* mRNA, which are key players in AD pathology and may promote inflammation and neurodegeneration, particularly in microglia, suggesting potential therapeutic targets for neuroinflammatory diseases. Research has revealed that miR-146a is intricately involved in cellular senescence and is associated with inflammatory senescence (Rippo et al., 2014). This kind of miRNA regulates key pathways that control both the aging of individual cells and the inflammatory response that typically accompanies this process. By influencing these pathways, miR-146a contributes to the broader inflammatory microenvironment that often accelerates age-related diseases, including neurodegenerative conditions. Sierksma et al. (2018) reported significant dysregulation of miR-146a in the AD brain and that Aβ or tau pathology drove these changes, confirming the potential of miR-146a as a biomarker. Moreover, Mai et al. (2019) demonstrated that a miR-146a agomir administered intranasally rescued pathological processes and cognitive deficits in a mouse model of AD. The results suggested that miR-146a may attenuate overall pathological processes, including neuroinflammation, Aβ deposition and tau phosphorylation, in an AD mouse model by targeting serine/arginine-rich splicing factor 6 (Srsf6). Liang et al. (2021b) reported that miR-146a modulates the microglial phenotype, reduces proinflammatory cytokines, enhances phagocytosis, and alleviates cognitive deficits in AD mice, suggesting potential therapeutic value.

**Figure 2:**
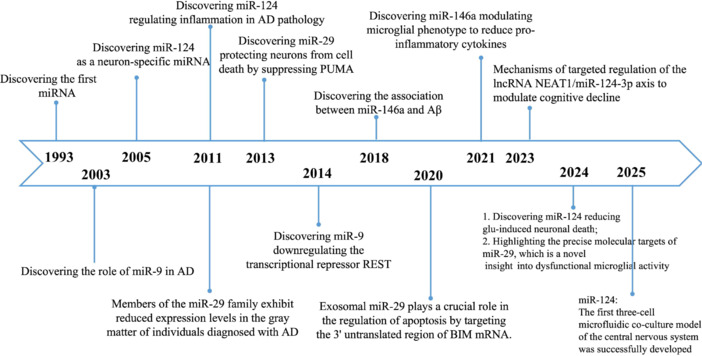
Milestones in the development of miRNAs in AD. AD: Alzheimer’s disease; Aβ: amyloid-β; BIM: Bcl-2 interacting mediator of cell death; lncRNA: long noncoding RNA; miRNA: microRNA; NEAT1: nuclear paraspeckle assembly transcript 1; PUMA: p53 upregulated modulator of apoptosis; REST: RE1 silencing transcription factor.

The miR-146 family comprises two pri-miRNAs, miR-146a and miR-146b, which play similar biological roles. The *miR-146* gene, which is responsible for encoding miR-146a, is located on human chromosome 5 (5q33.3) and mouse chromosome 11 (B1.1). A key characteristic of miR-146a is its remarkable conservation across mammalian species, highlighting its evolutionary importance (Mao et al., 2023). This conservation suggests that miR-146a plays crucial roles in various fields. By delivering miR-146a to mice with tumors, Chen et al. (2023) reported that miR-146a inhibited tumor growth by targeting immunosuppressive neutrophils and enhancing CD8^+^ T-cell infiltration. In nonobese diabetic mice, miR-146a-5p promoted beta cell dysfunction and apoptosis during inflammatory stress by inhibiting mitochondrial function (Krishnan et al., 2024). These findings suggest that miR-146a-5p is a potential therapeutic target for preserving beta cell integrity. A recent study identified miR-146a-3p as a critical regulator of Th17 cell differentiation (Duan et al., 2023). By regulating MBD2, a key protein involved in epigenetic regulation, miR-146a-3p effectively suppresses the differentiation of Th17 cells. This inhibition of Th17 differentiation reduces the inflammatory response in the lungs, thereby alleviating the severity of asthma. Microglia are resident immune cells in the central nervous system, and their dysfunction may lead to damage to neurogenesis. Fan et al. (2022) reported that miR-146a-5p targets Krüppel-like factor 4 (*Klf4*) mRNA through exosomal secretion from microglia, disrupting its regulatory role in neurogenesis, inhibiting spontaneous firing of excitatory neurons, and exacerbating symptoms of depression. As shown in **Figure 2**, the timeline fully illustrates the key nodes of these four miRNAs associated with AD. miRNAs play a vital role in AD, and the following sections of this review provide these mechanisms in AD pathology, emphasizing their potential as biomarkers and therapeutic targets.

## MicroRNAs Regulate Different Pathological Mechanisms of Alzheimer’s Disease

### miRNAs and amyloid-β clearance

#### Amyloid-β clearance and Alzheimer’s disease

The pathological hallmark of AD is the accumulation of Aβ. Amyloid plaque pathologies typically begin within the neocortex before progressively extending to adjacent homo-cortical regions, ultimately affecting the entire brain and disrupting normal brain function (Jucker and Walker, 2023). This progression aligns with the clinical manifestations observed in AD. Aβ is a protein fragment produced through the enzymatic cleavage of APP by β- and γ-secretases (Orobets and Karamyshev, 2023). The *APP* gene, situated on chromosome 21, encodes APP (Orobets and Karamyshev, 2023). It is broadly expressed *in vivo* and is more concentrated in neuronal tissues of the brain, with three major APP variations (APP695, APP751, and APP770). APP751 and APP770 are expressed in glial cells and support neurons, whereas APP695 is directly expressed in neurons (Goettemoeller et al., 2024). Aβ is produced through two pathways: the non-amyloidogenic pathway involving α-secretase, which does not lead to Aβ accumulation, and the amyloidogenic pathway mediated by β-secretase, which results in Aβ production (Delport and Hewer, 2022; Chen et al., 2024b). BACE1, a type I transmembrane protein and a key enzyme in Aβ synthesis, is upregulated in brain cells, especially in neurons, oligodendrocytes, and astrocytes (Bao and Shen, 2023; Coimbra et al., 2024). Genetically, mutations in the *PSEN1* and *PSEN2* genes, which disrupt γ-secretase activity and promote Aβ aggregation, are linked to early-onset AD, whereas the apolipoprotein E (*ApoE*) gene is the primary genetic risk factor for sporadic or late-onset AD (Yang et al., 2023). ApoE is the most common apolipoprotein in the brain, with three isoforms: ApoE2, ApoE3, and ApoE4 (Windham and Cohen, 2024). The three primary ApoE isoforms are distinguished by amino acid positions 112 and 158, which alternate between cystine and arginine (Lin and Holtzman, 2024). While all three isoforms are involved in Aβ clearance, ApoE4 is a less efficient lipid-transporter protein than the others (Raulin et al., 2022). Aβ oligomers contribute to AD progression by promoting tau propagation, neuronal hyperexcitability, and mitochondrial dysfunction. Studies involving transgenic mice show that amyloid deposition amplifies tau-related neuronal damage (Yi et al., 2024), while Aβ-induced hyperexcitability and reactive oxygen species production further exacerbate neurodegeneration (Dhapola et al., 2024).

#### miRNAs and amyloid-beta–related pathways

Different miRNAs influence Aβ-related pathways. In the brain, miR-485-3p, miR-22-3p, and miR-532-5p regulate CD36, SRY-box transcription factor 9 (SOX9), and EPHA4, respectively, to enhance phagocytosis and clearance mechanisms, thereby significantly reducing Aβ deposition (Koh et al., 2021; Xia et al., 2022; Liang et al., 2023b). miR-34a-5p, miR-125b-5p, miR-31, miR-338-5p, miR-186, and miR-200a-3p also specifically directly target *BACE1* mRNA, leading to a reduction in its expression and activity with decreased production of Aβ (Kim et al., 2016b; Qian et al., 2019; Wang et al., 2019; Barros-Viegas et al., 2020; Li et al., 2020). Several miRNAs, with various targets, have been also identified as key regulators of Aβ production and cognitive function in AD. miR-32533 targets cyclic adenosine monophosphate (cAMP)-responsive element binding protein 5 (*CREB5*) mRNA, which helps reduce cognitive impairment and Aβ overload (Zeng et al., 2025). Similarly, miR-98 suppresses hairy and enhancer of split (Hes)-related with YRPW motif protein 2 (HEY2), a positive regulator of the Notch signaling pathway, which, in turn, decreases Aβ production by inhibiting the activity of the Notch pathway (Chen et al., 2019a). Furthermore, miR-3940-5p regulates PSEN1, an essential component of the γ-secretase complex responsible for Aβ generation, thereby reducing Aβ production (Qi et al., 2024b). In contrast, some miRNAs such as miR-149-5p (lysine acetyltransferase 8/histone H4 lysine 16 acetylation), miR-17 (neighbor of *BRCA1* gene 1), and miR-33 (ATP binding cassette subfamily A member 1 (ABCA1)) may act as contributors to disease progression rather than as protectors, promoting Aβ production or accumulation and thereby contributing to AD pathology (Chen et al., 2020a; Estfanous et al., 2021; Tate et al., 2024).

#### The four miRNAs and amyloid-beta–related pathways

**Figure 3:**
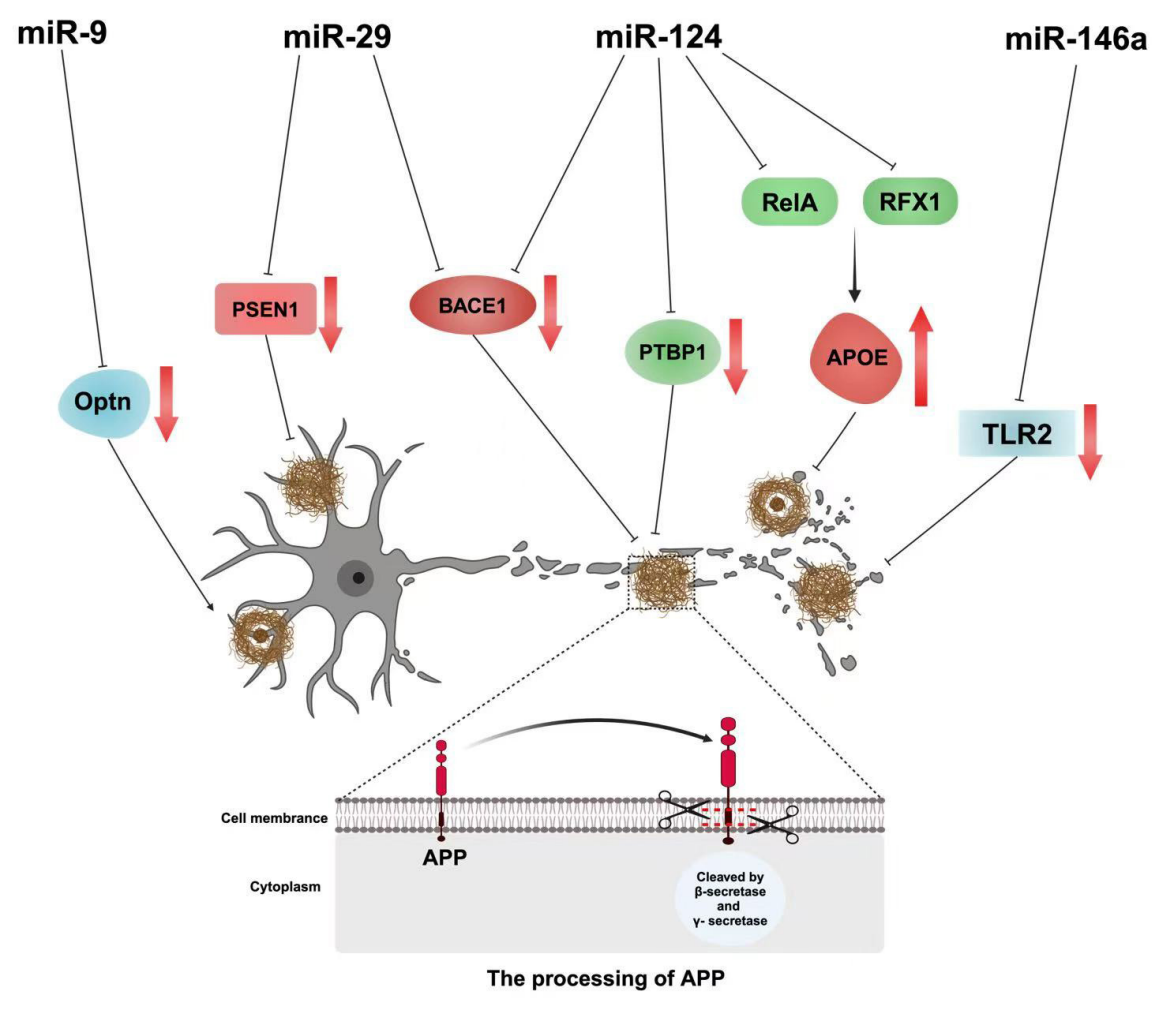
miRNAs regulate Aβ aggregation. In Aβ aggregation, miR-9 inhibits Optn to promote Aβ pathology. miR-29 inhibits PSEN1 and BACE1 to suppress Aβ production. miR-124 promotes APOE formation by inhibiting RelA and RFX1, which promotes Aβ peptide clearance and reduces Aβ deposition. miR-124 can also inhibit APP generation by inhibiting PTBP1, which reduces Aβ deposition. miR-124 can directly reduce BACE1 generation, which reduces APP miscutting and reduces Aβ deposition. Similarly, miR-146a reduces Aβ deposition by inhibiting TLR2. Created with BioRender.com. APOE: Apolipoprotein E; APP: amyloid precursor protein; Aβ: amyloid-β; BACE1: β-site APP-cleaving enzyme 1; miRNA: microRNA; Optn: optineurin; PSEN1: presenilin 1; PTBP1: polypyrimidine tract binding protein 1; RelA: the p65 subunit of nuclear factor kappa-B; RFX1: regulatory factor X1; TLR2: Toll-like receptor 2.

Importantly, miR-9, miR-29a, and miR-29b-1 were identified in the miRNA expression profiling of sporadic AD brain samples as potential regulators of *BACE1*, as they target its 3′-UTR. These findings suggest their role in modulating BACE1 expression in AD (Hébert et al., 2008). Studies conducted in different AD models have consistently shown that miR-29c-3p, which targets *BACE1*, reduces the accumulation of Aβ and attenuates neurotoxicity (Cao et al., 2021b; Wang et al., 2023c). Furthermore, for other members of the miR-29 family, Li et al. (2021a) reported that aluminum exposure in rat primary cortical neurons decreased miR-29a and miR-29b1 levels, potentially increasing BACE1 expression and contributing to amyloid deposit formation. On this basis, Sha et al. (2021) reported that bone marrow mesenchymal stem cell–derived extracellular vesicles deliver miR-29c-3p to inhibit BACE1 expression and activate the Wnt/β-catenin pathway, thereby exerting therapeutic effects in AD. Laminin receptor 1 pseudogene 1 (Lamr1-ps1), a pseudogene of the laminin receptor, significantly exacerbates early spatial learning and memory deficits in AD model mice, identifying the miR-29c/BACE1 pathway as a potential regulatory mechanism by which Lamr1-ps1 affects AD pathology and demonstrating the importance of the miR-29 family in regulating BACE1 (Wu et al., 2025). miR-124 has also been shown to bind the 3′-UTR of *BACE1* mRNA, suppressing its expression in neuronal models, which suggests a potential mechanism by which miR-124 could mitigate Aβ pathology (Fang et al., 2012). Autophagy plays a crucial role in mitigating Aβ deposition and slowing the progression of AD (Heckmann et al., 2019). In the early stages of AD, miR-9-5p is downregulated, whereas in the later stages, it becomes upregulated. This shift in miR-9-5p expression may contribute to the impairment of autophagy in the late stages of the disease. Notably, miR-9-5p regulates the autophagy receptor optineurin (Optn), further disrupting autophagic processes and potentially accelerating neurodegeneration (Chen et al., 2021). Furthermore, Mei et al. (2024) demonstrated that lowering miR-29a expression in the hippocampus of mice resulted in reduced Aβ deposition and a slowdown in cognitive decline, highlighting the potential therapeutic role of miR-29a modulation in AD. In addition, PSEN1, a key subunit of the γ-secretase complex, is regulated by miR-29b-2-5p, as shown by Wuli et al. (2022), which reduces Aβ plaque formation and improves cognitive function in mice. Furthermore, ApoE, which facilitates Aβ clearance, is also influenced by miR-124. miR-124-3p regulates the RelA protein, reducing its expression and thus enhancing ApoE-mediated Aβ clearance (Ge et al., 2020; Raulin et al., 2022). Additionally, the interaction of miR-124 with regulatory factor X1 and its role in promoting ApoE expression and function in microglia further underscore its potential in moderating Aβ dynamics (Feng et al., 2017). APP, an important aspect of Aβ generation, with exons 7 and 8, secretes more Aβ than those lacking these exons do (Donev et al., 2007). Polypyrimidine tract-binding protein 1 (PTBP1), along with its paralog PTBP2, regulates messenger RNA exon splicing. Conversely, miR-124 reduces PTBP1 levels while increasing PTBP2 (exclusive to the nervous system) to increase the jumping of APP exons 7 and 8, which reduces the production of Aβ (Smith et al., 2011; Zhu et al., 2020). miR-146a also plays a role in the regulation of Aβ. The expression of TLR2 has been found to have an inverse relationship with Aβ deposition. Zhang et al. (2015) reported that miR-146a can bind to the 3′-UTR of *TLR2* mRNA, thereby inhibiting its expression. This mechanism may explain the reduction in Aβ levels induced by miR-146a. Yang et al. (2021) reported that increased levels of miR-146a in microglia resulted in tolerance to Aβ/lipopolysaccharide stimulation, which in turn led to a reduction in Aβ clearance. Here, we present the relationship between miRNAs and Aβ clearance in **Figure 3**.

### miRNAs and tau protein

#### Tau protein and Alzheimer’s disease

Tau protein, which is known for its hydrophilic nature and stability under acidic and high-temperature conditions, is primarily encoded by the *MAPT* gene (Al Kabbani et al., 2025). This protein is characterized by four main regions: two structural domains (N-terminal and C-terminal) and multiple phosphorylation sites (Parra Bravo et al., 2024). Predominantly localized in axons, tau also appears in smaller quantities within neuronal dendrites and nuclei, influencing various cellular functions (Creekmore et al., 2024). In axons, tau stabilizes microtubules, enhances their assembly, and prevents their disassembly, which is crucial for axonal growth and maturation. In dendrites, tau is involved in regulating synaptic plasticity and contributing to the integrity of DNA and RNA in the nucleus (Zhang et al., 2024b). Neurofibrillary tangles, which consist of abnormally phosphorylated tau proteins, are another key pathological feature of AD. Many kinases contribute to the hyperphosphorylation of tau proteins. Initially, tau may be pre-phosphorylated by protein kinase A (PKA), protein kinase C (PKC), cyclin-dependent kinase 5 (CDK5), and calcium/calmodulin (CaM) kinase II, followed by further phosphorylation by GSK-3β, facilitating a smoother phosphorylation process (Telser et al., 2023; Cheng et al., 2024). In AD, neurofibrillary tangles first emerge in layer II of the entorhinal cortex and subsequently spread to the hippocampus and neocortex, reflecting the progressive neurodegenerative characteristics of the disease (Dutta et al., 2023; Walker et al., 2024). Pathological tau accumulation plays a crucial role in synaptic dysfunction and neuronal degeneration. For instance, the tau P301L transgenic mouse model rTg4510 has shown reductions in dendritic spine density and impairments in long-term potentiation, both of which are key indicators of synaptic plasticity (Santacruz et al., 2005). Moreover, tau-laden exosomes have been shown to be internalized by both local and distant cells, contributing to apoptosis and subsequent neuronal loss (Agosta et al., 2014). These findings suggest that tau pathology not only disrupts synaptic function but also facilitates the spread of toxicity throughout the brain, exacerbating neurodegeneration.

#### miRNAs and tau protein-related pathways

By organizing and further investigating the regulation of tau by miRNAs, we observed that miR-425-5p (heat shock protein B8) may act as an enhancer, promoting tau pathology (Yuan et al., 2020), while others may reduce the tau protein burden and mitigate neurodegeneration. miR-504-3p, miR-650, miR-483-5p, miR-132, and miR-148a-3p exhibit complementary roles in mitigating tau-associated pathology (Wang et al., 2017b; Nagaraj et al., 2021; Chen et al., 2022; Zeng et al., 2022b; Lin et al., 2023a). miR-504-3p targets *p39*; miR-650 modulates CDK5; miR-483-5p regulates extracellular signal-related kinase (ERK)1; miR-132 targets *NOS1* to decrease tau phosphorylation; and miR-148a-3p protects neuronal cells by targeting *p35*, preventing Aβ-induced tau hyperphosphorylation. Collectively, these miRNAs offer promising therapeutic avenues for addressing tauopathies and AD. Furthermore, a thorough understanding of the representative miRNAs may yield promising avenues for therapeutic intervention in AD.

**Figure 4:**
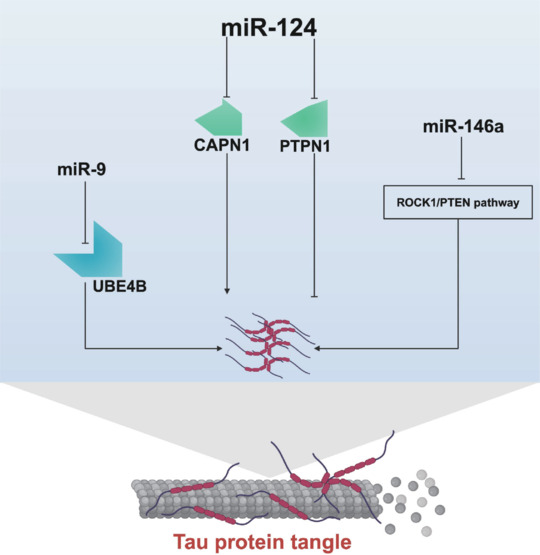
miRNAs regulate tau pathology. In the area of hyperphosphorylated tau protein, miR-9 directly inhibits UBE4B to promote tau hyperphosphorylation. miR-124 can inhibit CAPN1 and PTPN1 to regulate neurofibrillary tangle formation. miR-146a increases tau pathology through the ROCK1/PTEN pathway. Created with BioRender.com. CAPN1: Calpain1; miRNA: microRNA; PTEN: phosphatase and tensin homolog; PTPN1: protein tyrosine phosphatase nonreceptor type 1; ROCK1: Rho-associated coiled-coil kinase 1; UBE4B: ubiquitin conjugation E4B.

High levels of miR-124 in AD mice models have been suggested to reduce the expression of PTPN1, altering the phosphorylation dynamics of GSK-3 and protein phosphatase 2 (PP2A) and the overall balance of kinase/phosphokinase, and thereby contributing to the hyperphosphorylation of tau (Hou et al., 2020). However, other studies have reported different views on miR-124. Upregulation of miR-124 in AD brains has been associated with beneficial effects on these pathological processes. In addition to GSK-3, CDK5, a kinase expressed predominantly in neurons, has also been implicated in tau phosphorylation and plays an important role in neuronal development and migration. CDK5 activation leads to detrimental effects, including cell death, under pathological conditions (Allnutt et al., 2020). Although P35 is the main physiological CDK5 activator, calpain, a kind of enzyme highly expressed in neurons, can cleave p35 to p25. Thus, p25 can bind to CDK5 kinase and generate large amounts of CDK5/p25 complexes, leading to hyperphosphorylation of tau proteins (Maitra and Vincent, 2022). Earlier investigations have demonstrated that miR-124-3p diminishes calpain-1 expression, consequently reducing the proteolytic processing of p35 to p25. This decrease in p25 levels subsequently interferes with the assembly of the CDK5/p25 complex, potentially reducing the extent of tau hyperphosphorylation (Zhou et al., 2019). Through a comparative analysis of two studies, we observed that the choice of model system (APP/PS1 mice *versus* P301S mice) may significantly influence the direction of miR-124 regulation through distinct pathology-driven mechanisms (Aβ deposition-induced secondary tau phosphorylation *versus* mutant tau-triggered primary neurofibrillary tangles). Therefore, although miR-124 targets different genes, its bidirectional effect does not contradict its inherent function but instead results from the differential activation of target gene networks in distinct pathological contexts. These discrepancies in findings across different studies highlight the need for additional research to clarify the exact influence of miR-124 on tau pathology in AD. UBE4B, an E3/E4 ubiquitin ligase, clears tau proteins mainly through the ubiquitin-dependent autophagy-lysosomal system (ALS), a major protein degradation pathway in eukaryotic cells. Subramanian et al. (2021) demonstrated that miR-9a inhibits tau protein ubiquitin-dependent ALS-mediated autophagic clearance by inhibiting UBE4B. Increased expression of miR-146a has been shown to suppress the ROCK1/PTEN signaling pathway, which disrupts the normal regulation of tau phosphorylation, ultimately promoting tau hyperphosphorylation within neurons (Wang et al., 2016a). This may suggest that a better understanding of the molecular mechanisms by which miRNAs affect tau phosphorylation could offer novel therapeutic avenues for mitigating tau-related neurodegeneration. The relationship between miRNAs and hyperphosphorylated tau protein is summarized in **Figure 4**.

### miRNAs and neuroinflammation

#### Neuroinflammation in Alzheimer’s disease

Neuroinflammation is increasingly recognized as a critical factor in AD progression, playing a prominent role alongside Aβ plaques and neurofibrillary tangles (Liu et al., 2025; Zhang et al., 2025b). Recent advancements in positron emission tomography (PET) imaging agents targeting neuroinflammation have enabled noninvasive tracking of brain inflammatory events such as microglial activation and reactive astrocytes, which have advanced our understanding of AD (Chauveau et al., 2025). This inflammatory response is driven by microglia, astrocytes, and a variety of cytokines, along with key elements such as caspases, inflammasomes, chemokines, complements, and growth factors (Almutary et al., 2025). Tumor necrosis factor (TNF)-α, an essential cytokine for immune and neuronal function, contributes to a pro-inflammatory environment in the brain, exacerbating AD-like pathology and cognitive dysfunction (Wang et al., 2023b). More specifically, chronic inflammation sustains high TNF-α levels, stimulating Aβ synthesis, causing neuronal loss, and impairing microglial phagocytosis of Aβ and inducing APP and BACE1 expression in astrocytes (Plantone et al., 2023). Furthermore, the interleukin (IL) family, which includes IL-18, IL-33, IL-23, IL-12, IL-10, and IL-1, has been shown to influence the pathogenesis of AD (Chen et al., 2024d). Notably, IL-33 administration has been found to reverse synaptic plasticity impairments and memory deficits in APP/PS1 transgenic mice; this finding has been linked to a reduction in soluble Aβ levels and amyloid plaque deposition, likely due to the promotion of microglial recruitment and enhanced Aβ phagocytosis (Fu et al., 2016). Astrocytes from APP/PS1 mice have been reported to show excessive production of chemokines such as C–C motif ligand 2 (CCL2), C–X–C motif ligand 1 (CXCL1), and C–X–C motif ligand 10 (CXCL10) and elevated IL-6 levels upon IL-1β stimulation, contributing to the inflammatory response in AD (Lopez-Rodriguez et al., 2021).

In fact, acute and intense neuroinflammation can improve the clearance of AD pathological features. However, persistent inflammation can be harmful. IL-1β, a potent pro-inflammatory cytokine, is notably elevated in the brains of patients with AD. Ghosh et al. (2013) found that acute and strong IL-1β stimulation in APP/PS1 mice enhanced the clearance of Aβ, while this beneficial pro-inflammatory factor became harmful in chronic inflammation in transgenic mice. The inflammasome is a complex of multiple proteins that play key roles in triggering inflammatory responses. In AD, the NLRP3 inflammasome is particularly relevant, since it is activated and its expression is upregulated. The activation of NLRP3 inflammasomes promotes the aggregation and hyperphosphorylation of tau proteins as well as the deposition of Aβ (Wang et al., 2024b). These events exacerbate long-term potentiation impairments and spatial memory deficits in APP/PS1 transgenic mice, a commonly used model for AD. Targeting TNF-α or its signaling pathways may represent a promising strategy for mitigating the inflammatory component of AD. Sedighi et al. (2019) found that miR-29a significantly reduced the level of tumor necrosis factor-α (TNF-α) and thus attenuated the inflammatory response, possibly by targeting the TNF-mediated pathway or the TNF-α receptor 1 (TNFR1). CFH is a key regulator in the complement cascade that acts as an inhibitor of excessive inflammatory responses. However, upregulation of miR-146a has been demonstrated to decrease CFH expression, which may have significant implications for the regulation of neuroinflammation (Lukiw et al., 2008). The reduced levels of CFH could lead to exacerbation of inflammatory processes, potentially contributing to the progression of neurodegenerative diseases such as AD. Likewise, chronic inflammatory conditions such as obesity and diabetes can contribute to AD progression primarily by activating microglia and astrocytes (Rohm et al., 2022). Therefore, the following sections will focus on the specific roles of microglia and astrocytes in the neuroinflammation associated with AD.

#### miRNAs and microglia

*Microglia and neuroinflammation*

Microglia, first identified in 1919, are small neuroglial cells that act as the resident macrophages of the central nervous system (CNS), monitoring brain regions under their jurisdiction and preventing pathogen invasion or debris accumulation. Microglial activation plays a key role in neuroinflammation in AD (Wang et al., 2023a). Microglia show two distinct morphologies after being stimulated: M1 microglia show a pro-inflammatory phenotype characterized by elevated levels of pro-inflammatory cytokines and secretion of pro-inflammatory cytokines such as TNF-α and members of the IL family. In contrast, M2 microglia exhibit an anti-inflammatory phenotype characterized by the secretion anti-inflammatory cytokines. These two phenotypes are not permanent, and microglia can switch between the M1 and M2 phenotypes in response to different stimuli, a process known as “alternative activation” (Song and Suk, 2017; Antignano et al., 2023). In AD, Aβ and injured neurons serve as primary ligands for microglial activation and bind to immune pattern-recognition receptors on the surface of microglia (Fernando and Wijayasinghe, 2021). These pattern-recognition receptors include receptors like TLR, the receptor for advanced glycosylation end-products, NOD-like receptors, scavenger receptors, and receptors such as CD36, CD14, and CD47 (Castro-Gomez and Heneka, 2024).

*miRNAs and microglia-related pathways*

Some miRNAs act on microglia to regulate the inflammatory response in AD. miR-155 targets signal transducer and activator of transcription 1 (*STAT1*) mRNA and interferon-γ (IFN-γ) signaling to reduce microglial acquisition of a neurodegenerative phenotype, and miR-181c-5p enhances microglial phagocytosis of Aβ by targeting P38, thereby mitigating neuroinflammation in AD (Yin et al., 2023; Li et al., 2024). Furthermore, miR-25802 promotes the polarization of microglia to a pro-inflammatory M1 phenotype through Klf4/NF-κB signaling, contributing to inflammatory responses in neurodegeneration (Zhao et al., 2024).

miR-124, a specific miRNA, has been shown to modulate microglial activation and has promising implications for AD-related neuroinflammation. Specifically, miR-124-3p in microglial exosomes inhibits the *RelA* gene, which encodes the p65 subunit of NF-κB, thereby promoting anti-inflammatory M2 polarization in microglia and reducing the possibility of AD secondary to repetitive mild traumatic brain injury (Ge et al., 2020). This classical pathway is activated when inflammatory stimuli release inhibitor of NF-κB (IκB) from the NF-κB complex, allowing NF-κB, including the p50 and p65 (Rel-A) subunits, to enter the nucleus and initiate gene transcription (Liu et al., 2017b). TLR4 is abundant in microglia and promotes inflammatory cytokine production. Yang et al. (2022) demonstrated that miR-124 downregulates several components of this pathway, including TLR4, myeloid differentiation primary response gene 88 (MyD88), NF-κB p65, and NLRP3, thereby reducing M1 microglial activation. Additionally, miR-124 increases the expression of the TLR4 co-receptor CD14, which shifts signaling toward anti-inflammatory responses, including the production of interferon-β (IFN-β), by activating Toll-like receptor adaptor molecule (TRAM)/Toll-interleukin-1 receptor (TIR)-domain-containing adaptor inducing interferon-β (TRIF) signaling in microglia (Kagan et al., 2008; Zanoni et al., 2011; Pajarskienė et al., 2021). IFN-β, a key type I interferon secreted by microglia, facilitates the clearance of myelin debris in the CNS and reduces the expression of inflammatory factors including IL-6, IL-8, and TNF-α (Kocur et al., 2015). Moreover, miR-124 in neurons and microglia inhibits p38 expression by inhibiting the p38/mitogen-activated protein kinase (MAPK) signal pathway, which is upstream of NF-κB signaling pathway, and further decreasing IL-6 and IL-1β expression to reduce neuroinflammation (Lawson et al., 2013; Yao et al., 2019a). Beyond miR-124, other pri-miRNAs act at different sites in the NF-κB signaling pathway to influence neuroinflammation in AD. miR-9 promotes microglial activation by targeting the monocyte chemotactic inducing protein 1 (MCPIP1)/NF-κB pathway (Yao et al., 2014). In addition, overexpression of miR-29a-3p has been shown to decrease phagocytosis of lipopolysaccharide-stimulated BV-2 cells, while its inhibition increases phagocytic activity, highlighting the regulatory role of miR-29a-3p in microglial function (Scoyni et al., 2024). miR-146a can effectively inhibit microglial activation in the hippocampus of AD model mice. Specifically, miR-146a suppresses the expression of key inflammatory genes associated with this pathway, such as IRAK-1, tumor necrosis factor receptor-associated factor 6 (TRAF6), and NF-κB. These molecules play central roles in the activation of downstream inflammatory processes. By inhibiting their expression, miR-146a reduces the production and release of pro-inflammatory cytokines, including IL-1β, TNF-α, and IL-6, and improves cognitive function in mice (Mai et al., 2019). Other studies have shown that miR-146a is upregulated in AD through regulation of NF-κB. miR-146a upregulation leads to a decrease in the expression of IRAK-1 and an increase in the expression of IRAK-2, and the upregulation of IRAK-2 may promote microglial activation in response to chronic inflammation in AD (Cui et al., 2010). Mature microglia are derived from primitive macrophages that differentiate in the mammalian yolk sac before blood circulation begins (Ginhoux et al., 2010). CCAAT/enhancer-binding protein-α (C/EBPα) and purine-rich box 1 (PU.1), two key regulators in early myeloid development, are essential for yolk sac microglial generation; neutrophils and microglia are absent in PU.1- and C/EBPα-deficient mice (Beers et al., 2006; Lawrence et al., 2018). miR-124 targets *C/EBPα* and *PU.1* mRNAs and downregulates the expression of granulocyte-macrophage colony–stimulating factor receptor, CD11b, CD45, F4/80, and major histocompatibility complex class II to reduce the activation of bone marrow-derived macrophages and splenic macrophages. Meanwhile, miR-124 downregulates M1-related markers and upregulates M2-related markers such as transforming growth factor (TGF)-β1, which are found in inflammatory zone 1, and arginase 1; thus, miR-124 promotes a surveillant phenotype among microglia by regulating the expression of C/EBPα and activating PU.1 (Ponomarev et al., 2011, 2013). Recent research suggests that microglia are the most dynamic cells in the brain, constantly responding in different ways (by retracting and extending their branches) to changes in their surrounding environment (neighboring tissue cells and their secretion of various factors) without abruptly switching from “resting” to “activated” states as a result of other pathological conditions (Nimmerjahn et al., 2005; Hanisch and Kettenmann, 2007). Therefore, some scholars have renamed “resting” microglia as “surveying” or “surveillant” (Paolicelli et al., 2022). However, many scholars still believe in the idea that microglia are referred to as “resting” under physiological conditions and “activated” in their reactive form under pathological conditions; in this paper, we have used “surveillant” to support the new perspective. To summarize, miRNAs regulate neuroinflammation in AD through multiple mechanisms of modulation of activated microglial activity, while miR-124 can also keep microglia in a surveillant state, mitigating neuroinflammation in another way.

#### miRNAs and astrocytes

*Astrocytes and Alzheimer’s disease*

Astrocytes are the largest and most abundant glial cells in the CNS and play essential roles in maintaining the integrity of the blood‒brain barrier and ion homeostasis (Wang et al., 2025). They regulate ionic and fluid balance across cell membranes, with Kir4.1 channels facilitating extracellular potassium (K^+^) buffering and influencing cerebral blood flow and the blood‒brain barrier by releasing signaling molecules such as prostaglandins and nitric oxide (Baldwin et al., 2024; Liddelow et al., 2024; Rangel-Gomez et al., 2024). Chronic neuroinflammation results in irreversible neuron loss, thus impairing memory and cognitive function in AD. As neurons are nonrenewable, reprogramming nonneuronal cells, such as astrocytes, into neurons represents a promising therapeutic strategy for neurodegenerative conditions (Baranes et al., 2023). Astrocytes are activated into reactive astrocytes because of various stimuli, which have certain stem cell properties. Research has shown that transcription factors such as NeuroD1, neurogenin-2, and distal-less homeobox 2 can induce the reprogramming of reactive astrocytes into neurons (Heinrich et al., 2010; Shimada et al., 2012; Sirko et al., 2013).

*miRNAs and astrocyte-related pathways*

**Figure 5:**
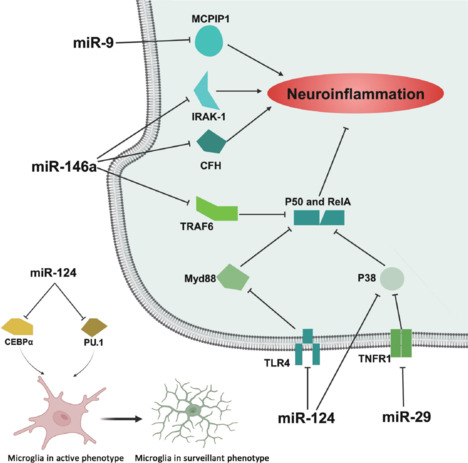
miRNAs modulate neuroinflammation. The inhibition of MCPIP1 expression by miR-9 promotes neuroinflammation. miR-29 reduces neuroinflammation through the NF-κB pathway by inhibiting TNFR1, which in turn continues to inhibit P38. The inhibition of IRAK-1 and CFH by miR-146a promotes the exacerbation of neuroinflammation, and in addition, miR-146a may reduce neuroinflammation by inhibiting TRAF6 and participating in the TRAF/NF-κB pathway. The effects of miR-124 on microglial activation can be divided into two signaling pathways, the TLR4/Myd88/NF-κB signaling pathway and the p38/MAPK/NF-κB signaling pathway, which ultimately lead to a decrease in M1-type microglia and an increase in M2-type microglial polarization. Furthermore, miR-124 inhibits C/EBPα and PU.1 to promote a surveillance phenotype in microglia. Created with BioRender.com. CD14: Cluster of differentiation 14; CFH: complement factor H; C/EBPa: CCAAT/enhancer-binding protein-α; IRAK-1: interleukin-1 receptor associated kinase 1; MAPK: mitogen-activated protein kinase; MCPIP1: monocyte chemotactic inducing protein 1; miRNA: microRNA; Myd88: myeloid differentiation primary response gene 88; NF-κB: nuclear factor kappa-B; PU.1: purine-rich box 1; TLR4: Toll-like receptor 4; TNFR1: tumor necrosis factor receptor 1; TRAF6: tumor necrosis factor receptor-associated factor 6; TRAM: Toll-like receptor adaptor molecule; TRIF: Toll-interleukin-1 receptor-domain-containing adaptor inducing interferon-β.

miRNAs, particularly miR-124, play a critical role in this reprogramming process. For example, miR-124 has been shown to promote the conversion of astrocytes into neurons and ameliorate nerve injury by inhibiting the expression of Delta-like 4 (DLL4) and Notch1 (Jiao et al., 2017; Guo et al., 2023). Ezh2 (a histone methyltransferase) is downregulated by miR-124 to promote neuronal growth and counteract astrocyte-specific differentiation (Neo et al., 2014). In addition, miR-124 reduces the protein expression of SOX9, nuclear factor I A (NFIA), and hairy and enhancer of split 1 (HES1), and increases the expression of Achaete-scute family bHLH transcription factor 1 (Ascl1), which is associated with neuronal differentiation, promoting the differentiation of astrocytes into neurons (Zheng et al., 2021b). The interaction between microglia and astrocytes in neuroinflammation is an emerging area of interest, especially in AD. In a mouse model of stroke, astrocytes exposed to M2 microglial extracellular vesicles enriched with miR-124 exhibited decreased Notch1 expression and increased SRY-related HMG-box transcription factor 2 (SOX2) expression, which reduced astrocyte proliferation and glial scarring and promoted astrocyte transformation into neuronal progenitor cells, contributing to neuronal regeneration (Li et al., 2021b). However, the role of miRNAs in mediating interactions between microglia and astrocytes in AD remains underexplored, presenting a potential new avenue for research on inflammation. A schematic diagram of miRNAs regulating neuroinflammatory mechanisms is presented in **Figure 5**.

### miRNAs and synaptic dysfunction

#### Synaptic dysfunction and Alzheimer’s disease

Synapses are indispensable structures in neuronal cells that facilitate communication and information transfer between neurons (Attardo and Cambridge, 2024). In the human brain, most are chemical synapses, which consist of a presynaptic component, a synaptic cleft, and a postsynaptic component. When the nerve impulse reaches the presynaptic membrane, the calcium concentration in the presynaptic membrane increases, triggering the fusion of synaptic vesicles with the presynaptic membrane and the release of neurotransmitters into the synaptic cleft via cytosolic release (Südhof, 2021). The neurotransmitters then diffuse to the postsynaptic membrane, where they bind to receptors on the membrane, including fast ligand–gated ion channels or slow G-protein receptors, which trigger the opening or closing of the channels, thus transmitting nerve impulses (Südhof, 2021). In addition, the mature function of synapses is crucial for memory consolidation and formation. Synapses are critical for the phenomenon of persistent enhancement in signaling between two neurons, and intact information transmission can continuously stimulate both types of neurons, thereby increasing learning and memory, which is also known as long-term potentiation (Kruijssen and Wierenga, 2019). Unfortunately, synaptic loss is found in the early stages of AD and cognitive dysfunction, especially in the hippocampus; however, this alteration can either occur before the onset of amyloid plaques and neurofibrillary tangles or be continued by their deterioration (Chen et al., 2019b).

#### miRNAs and synapsis-related pathways

The role of miRNAs in synaptic function in AD requires attention, and aberrant expression of some specific miRNAs has been reported to lead to synaptic dysfunction. In miR-455-3p-deficient mice, mitochondrial biogenesis, dynamics, and synaptic activity were shown to be significantly impaired, and miR-431 was found to regulate SMAD family member 4 (SMAD4) to attenuate synaptic damage, thus protecting synaptic integrity and neuronal function (Kumar et al., 2021; Ge et al., 2023). miR-30b regulates key proteins, including ephB2, sirtuin 1, and GluA2, while miR-134-5p regulates plasticity-related proteins, cAMP-response-element binding protein 1 (CREB-1), and brain-derived neurotrophic factor (BDNF), disrupting synaptic plasticity and memory function by downregulating these proteins (Song et al., 2019; Baby et al., 2020). Furthermore, miR-429-3p and miR-181a regulate mitogen-activated protein kinase phosphatase 1 and GluA2, respectively, to negatively regulate synaptic plasticity (Rodriguez-Ortiz et al., 2020; Luo et al., 2024). The miR-188-5p level has been shown to be reduced in brain tissue from both patients with AD and 5xFAD mice, whereas increasing the miR-188-5p level was shown to ameliorate synaptic dysfunction in an AD mouse model (Lee et al., 2016).

**Figure 6:**
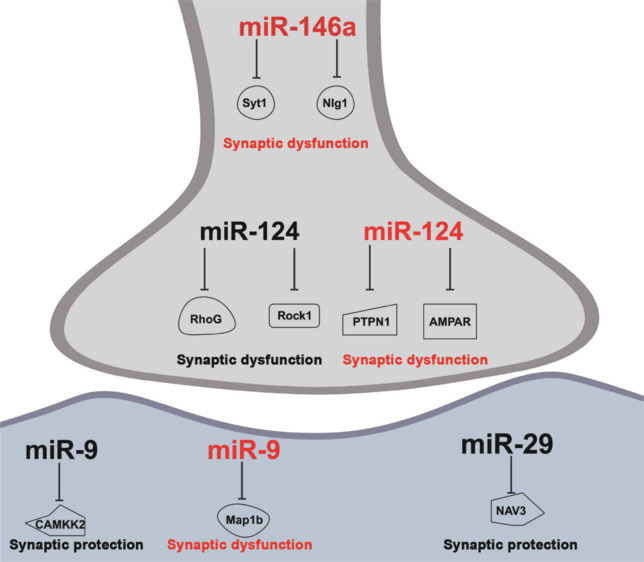
miRNAs modulate synaptic function. First, miR-9 regulates synaptic function through the inhibition of CAMKK2 and Map1b. miR-29 achieves synaptic protection by inhibiting NAV3. miR-146a inhibits both Syt1 and Nlg1 to disrupt synaptic plasticity as well as synaptic function. miR-124 can promote the structural growth of synapses by inhibiting RhoG and ROCK1. However, miR-124 can inhibit AMPAR and suppress PTPN1, resulting in reduced synaptic transmission. Created with BioRender.com. AMPAR: α-Amino-3-hydroxy-5-methyl-4-isoxazole propionic acid receptor; CAMKK2: Ca^2+^/calmodulin kinase kinase protein 2; MAP1b: microtubule-associated protein 1B; miRNA: microRNA; NAV3: neuron navigator 3; Nlg1: neuroligin1; PTPN1: protein tyrosine phosphatase nonreceptor type 1; RhoG: Ras homolog family member G; ROCK1: Rho-associated coiled-coil kinase 1; Syt1: synaptotagmin1.

miR-29b positively regulates axon guidance and synapse formation in neuronal cells by binding to the 3′untranslated region (UTR) of neuron navigator 3 (*NAV3*) mRNA and inhibiting *NAV3* translation, thereby reducing NAV3 protein expression. Moreover, miR-146a inhibits the expression of presynaptic Syt1 and postsynaptic Nlg1, which play critical roles in dendritic spine formation and synaptic stability, thereby impairing synaptic function (Prada et al., 2018; Jahangard et al., 2020). Chang et al. (2014) found that overexpression of miR-9 can restore Aβ_42_-induced dendritic spine loss by attenuating the Ca^2+^/calmodulin kinase kinase protein 2 (CAMKK2)-AMPK pathway. Endogenous Aβ accumulation within human neurons has been shown to initiate substantial alterations in dendritic mitochondrial dynamics, resulting in both structural remodeling and a reduction in mitochondrial biomass. This process is accompanied by hyperactivation of AMPK. Interestingly, pharmacological inhibition of CAMKK2 or direct suppression of AMPK has demonstrated protective effects on hippocampal neurons, safeguarding them from the synapse degeneration induced by oligomeric Aβ_42_ species (Lee et al., 2022). In addition, Li et al. (2016) observed that osthole upregulates miR-9 expression and protects against neural loss through the same pathway. Microtubule-associated protein 1B (Map1b) belongs to an important class of proteins for microtubule stability. Past studies have reported that miR-9 inhibits *Map1b* translation and reduces axon length and promotes branching, affecting microtubule stabilization and ultimately leading to synaptic dysfunction (Dajas-Bailador et al., 2012). This finding underscores the intricate role of miR-9 in modulating synaptic function. Additional research is required to elucidate the specific mechanisms through which miR-9 influences synaptic dynamics and to better understand its potential as a therapeutic target in neurodegenerative diseases. miR-124 performs multiple regulatory activities in synaptic function. miR-124 promotes synaptogenesis by suppressing ROCK1 expression and activating the PI3K/Akt signaling cascade, thereby facilitating synaptic development and maturation (Gu et al., 2014). Moreover, miR-124 promotes neurite growth by decreasing the expression of RhoG, a small GTPase, inhibits the expression of Rac1 through the engulfment and cell motility (ELMO)/Dock180/Rac1 signaling pathway, and directly suppresses Cdc42, resulting in axonal and dendritic growth (Franke et al., 2012). Glutamate, which is highly abundant in brain, belongs to the most basic class of neurotransmitters that transmit information in the nervous system. Accumulation of glutamate in the extracellular space triggers overactivation of glutamatergic receptors and leads to excitotoxicity. miR-124-3p transferred from neuronal exosomes to astrocytes has been shown to counteract the effects of miR-132 and miR-218, leading to an increase in GLT-1 expression in astrocytes, which prevents excitotoxicity by clearing excess glutamate around synapses (Men et al., 2019). α-Amino-3-hydroxy-5-methyl-4-isoxazole propionic acid receptor (AMPAR) is a receptor for glutamate and mediates rapid excitatory synaptic transmission in the CNS. Conversely, one study showed that in an AD mice model, miR-124-3p downregulates PTPN1 and AMPAR expression to damage synaptic transmission, ultimately resulting in neuronal death (Wang et al., 2018). These discrepant roles of miR-124 in AD may stem from multiple fundamental differences in microenvironment regulatory mechanisms across experimental models. First, the studies by Franke et al. (2012) and Gu et al. (2014) used *in vitro* brain tissue models to validate the target interactions of miR-124 but failed to recapitulate the dynamic remodeling of cerebral microenvironments induced by the progression of AD-related factors such as Aβ. Second, *in vivo* models differ notably among studies: While Men et al. (2019) utilized CD63-GFP^f/f^ knock-in mice lacking characteristic AD pathological phenotypes, Wang et al. (2018) used Tg2576 transgenic with definitive Aβ deposition, which influenced the evaluation of the effects of AD-specific pathological microenvironments on miR-124 regulatory networks. Moreover, the levels of miR-124 varied across most experiments, and the study by Wang et al. (2018) failed to capture the dose-dependent nature of miR-124. This observed variability strongly indicates that the functional impact of miR-124 in AD pathogenesis exhibits concentration-dependent characteristics, potentially mediating distinct molecular pathways at different expression thresholds. Likewise, *PTPN1* mRNA is also targeted by miR-124 to cause tau hyperphosphorylation, another pathological feature of AD. Coincidentally, miR-124 levels were shown to increase in two experimental models, which may explain its negative role in AD. Furthermore, analyses of samples from patients with AD have revealed differences in miR-124 expression across various brain regions (cortex and hippocampus). Brites (2020) concluded that in postmortem samples from patients with Braak stage III disease, miR-124 expression was significantly upregulated throughout the hippocampal fissure, whereas Smith et al. (2011) observed a decrease in miR-124 expression in the anterior temporal cortex. This discrepancy may stem from variations in assay methodologies and sample-processing techniques. Differences in postmortem delay times may also influence the extent of RNA degradation, particularly affecting miRNA stability. Furthermore, pharmacological treatments administered during a patient’s lifetime may selectively modulate miRNA expression in distinct brain regions. Disease stage may also contribute to regional variations in miR-124 expression. Lukiw (2007) reported a reduction in the miR-124 levels in the hippocampus, which may be attributable to the pathological progression of AD and the associated loss of compensatory mechanisms. Altogether, these mechanisms imply that miRNAs offer substantial great potential for modulating synaptic functions. A schematic representation of miRNAs and synaptic dysfunction is presented in **Figure 6**.

### miRNAs and neurons

#### Neuronal death and Alzheimer’s disease

Apoptosis, the programmed cell death response to certain stimuli, is a natural process essential for cellular turnover (Gao et al., 2024; Tang et al., 2024). Imbalances in apoptosis can lead to various diseases: decreased apoptosis is linked to cancer, whereas excessive apoptosis contributes to neurodegenerative disorders, including AD (Takikawa et al., 2025). In AD, neuronal apoptosis primarily affects the hippocampus and cerebral cortex, resulting in impaired memory and cognitive functions. This neuronal apoptosis can arise from neuroinflammation, Aβ accumulation, tau protein tangles, mitochondrial dysfunction, and cholinergic deficits. The expression of multiple genes plays a role in neuronal apoptosis in AD. For example, reduced Bcl-2 levels in the hippocampus facilitate neuronal apoptosis, whereas BAX and BAK promote the release of cytochrome c, resulting in the activation of caspase-9 and initiating an apoptotic cascade. The Bcl-2/BAX ratio is crucial, with higher Bcl-2 suggesting cell survival and elevated BAX indicating a predisposition to cell death (Balusu and De Strooper, 2024; Zhang et al., 2024c).

#### miRNAs and neuronal death–related pathways

miRNAs play a significant role in regulating neuronal apoptosis. miR-30a-5p and miR-153-3p suppress the MAPK/NF-κB pathway in microglia and the ERK/signal transducer and activator of transcription 3 (STAT3) pathway in astrocytes, reducing lipopolysaccharide-induced neuronal death (Choi et al., 2022). miR-277 targets the mRNA of the head involution defective gene to reduce neuronal death, while miR-34a alleviates Aβ-induced SH-SY5Y cell death by regulating caspase-2 (Li et al., 2019b; Deshpande et al., 2024). Additionally, miR-193a-3p regulates PTEN, attenuating Aβ-induced neurotoxicity and apoptosis (Cao et al., 2020). Other miRNAs, including miR-133b (epithermal growth factor receptor), miR-383-5p (kinesin family member 1B), miR-148a-3p (ROCK1), miR-381-3p (prostaglandin-endoperoxide synthase 2), and miR-193a-3p (PTEN), effectively attenuate Aβ-induced apoptosis, offering potential therapeutic strategies for neurodegenerative diseases by protecting neurons from death (Yang et al., 2019; Cao et al., 2020; Zhang et al., 2021a, 2022; Li et al., 2022). miR-16-5p, miR-138, and miR-142 regulate Bcl-2, DEK oncoprotein, and brain angiogenesis inhibitor 3 to promote neuronal apoptosis, respectively (Kim et al., 2020; Miao et al., 2020; Fu et al., 2021). miR-351-5p inhibits Miro2, leading to disruption of mitochondrial trafficking, unbalanced mitochondrial fission, and excessive mitochondrial autophagy, and contributing to mitochondrial dysfunction, culminating in neuronal death and neurodegeneration (Woo et al., 2021).

miR-9-5p overexpression inhibits Aβ 25-35-induced mitochondrial dysfunction and apoptosis by reducing GSK-3β expression in HT22 cells (Liu et al., 2020a). Using Aβ-treated neuroblastoma cells, Liu et al. (2020b) observed that miR-29c-3p attenuated Aβ-induced neuronal death by regulating the NF-κB signaling pathway through direct regulation of tumor necrosis factor-α-inducible protein-1 (TNFAIP1). Bcl-2 interacting mediator of cell death (BIM), a BH3-only protein, plays a critical role in initiating apoptosis by activating Bcl-2-like protein 4 (BAX), a key effector protein in the mitochondrial apoptosis pathway (Czabotar et al., 2009; Xu and Ye, 2022). Jahangard et al. (2020) observed that miR-29b exerts a regulatory influence on apoptosis by inhibiting the translation of *BIM*. This inhibition occurs when miR-29b binds to the 3′-UTR of *BIM* mRNA, leading to a reduction in BIM protein expression. miR-146a plays a pivotal role in regulating neuronal death through a complex mechanism. The downregulation of miR-146a can influence various cellular signaling pathways in AD. Specifically, decreased levels of miR-146a may cause inhibition of the STAT1/myelocytomatosis oncogene (MYC) pathway and activation of the low-density lipoprotein receptor-related protein-2 (Lrp2)/Akt pathway, which together contribute to reducing apoptosis in AD, highlighting the multifaceted role of miR-146a in neuronal survival (Zhang et al., 2016; Ma et al., 2021). Furthermore, the inflammatory signaling pathway NF-κB induced upregulation of miR-146a-5p promoted hippocampal neuronal oxidative stress and pyroptosis by targeting downregulation of TP53-induced glycolysis and apoptosis regulator (*TIGAR*) (Lei et al., 2021). In AD, miR-124 also regulates neuronal death. For instance, syringin-enhanced miR-124-3p expression has been shown to reduce BH3-interacting domain death agonist (BID) expression and mitigate Aβ-induced neurotoxicity by blocking apoptosis (Zhang et al., 2021c). DAPK1 is a serine/threonine protein kinase that relies on Ca^2+^/calmodulin for activation. One of its critical functions is phosphorylating the downstream substrate NR2B. Additionally, the activation of the DAPK1/N-myc downstream-regulated gene 2 (NDRG2) signaling pathway has been implicated in promoting neuronal death, a hallmark of AD (Kim et al., 2016a; Shu et al., 2016). Shi et al. (2022b) observed that in mouse traumatic brain injury models, miR-124 specifically binds to and decreases the level of DAPK1, subsequently downregulating the DAPK1/NDRG2 pathway and decreasing neuronal death. This finding may indicate a potential application of miR-124 for neuronal apoptosis in AD. In models of glutamate-induced neuronal injury, miR-124-3p, which is present within exosomes from M2-polarized microglia, has been shown to suppress the expression of ROCK and phosphorylated phosphatase and tensin homolog (P-PTEN) while increasing the levels of phosphorylated Akt and phosphorylated mTOR, thereby reducing neuronal apoptosis through the ROCK/PTEN/Akt/mTOR signaling pathway (Zhu et al., 2024). In summary, miRNAs modulate multiple pathways associated with neuronal survival in AD, highlighting their potential as therapeutic targets to mitigate apoptosis and preserve neuronal function.

#### Neuronal development and Alzheimer’s disease

The two previously mentioned pathological features, Aβ and tau proteins, ultimately lead to neuronal death in AD. Tau proteins, which are abundant in axons, are crucial for microtubule assembly and stability, and are found in smaller amounts in dendrites, where they support synaptic plasticity by modulating the dendritic cytoskeleton (Rumpf et al., 2019). Abnormally phosphorylated tau protein affects axonal stability and neuronal development, thereby inducing neuronal death and brain dysfunction (Ye et al., 2024). Furthermore, Aβ may exacerbate tau hyperphosphorylation, potentially leading to cognitive impairments (Welikovitch et al., 2025). Therefore, strategies that promote neuronal maturation could counteract the damage caused by AD. Regulating Janus kinase 2 (JAK2) and checkpoint kinase 2 (CHEK2) by miR-433 and miR-669b-5p, respectively, has been shown to increase neuronal cell survival viability and proliferation (Wang and Zhang, 2020; A et al., 2023).

#### miRNAs and neuronal development–related pathways

**Figure 7:**
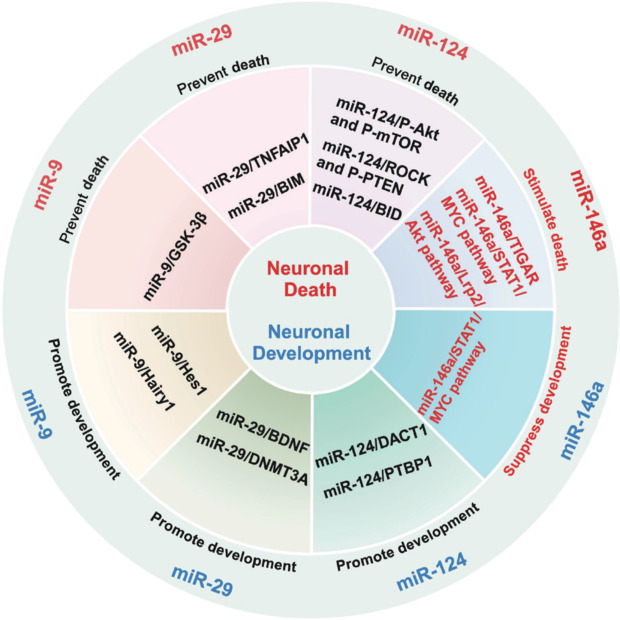
Relationships between miRNAs and neuronal death and development. miR-9 regulates GSK-3β, HES1, and hairy1 to prevent neuron death and promote neuron development. miR-29 directly targets TNFAIP1 and inhibits the translation of BIM to attenuate Aβ-induced neuronal death. By targeting DNMT3 and BDNF expression, miR-29 promotes neuronal proliferation and development. miR-124 decreases DAPK1 and BID, whereas miR-124 decreases ROCK and PTEN, elevates Akt and mTOR through the ROCK/PTEN/Akt/mTOR pathway, and ultimately reduces neuronal apoptosis. In addition, miR-124 represses DACT1 and regulates PTBP1 to promote neuron development. miR-146a promotes neuronal death and inhibits neuronal development through the STAT1/MYC pathway and the LrP2/Akt pathway. miR-146a-5p promotes hippocampal neuronal oxidative stress and pyroptosis by downregulating TIGAR. Created with BioRender.com. Akt: Protein kinase; BDNF: brain-derived neurotrophic factor; BID: BH3 interacting domain death agonist; BIM: Bcl-2 interacting mediator of cell death; DACT1: dishevelled binding antagonist of beta-catenin 1; DAPK1: death-associated protein kinase 1; DNMT3: DNA methyltransferase 3A; GSK-3β: glycogen synthase kinase-3β; HES1: hairy and enhancer of split 1; LrP2: low-density lipoprotein receptor-related protein-2; miRNA: microRNA; mTOR: mechanistic target of rapamycin; MYC: myelocytomatosis oncogene; PTEN: phosphatase and tensin homolog; PTBP1: polypyrimidine tract-binding protein 1; ROCK: Rho-associated coiled-coil forming protein kinase; STAT1: signal transducer and activator of transcription 1; TIGAR: TP53-induced glycolysis and apoptosis regulator; TNFAIP1: tumor necrosis factor-α-inducible protein-1.

miR-206-3p enhances neuronal morphology, increases Nissl body formation, and protects neurons from damage by increasing BDNF expression, which supports neuronal survival and synaptic plasticity, whereas miR-211-5p (NUAK1) and miR-409-5p (Plek) impair neurite growth and reduce neuronal survival and viability, highlighting the importance of miRNAs in both the development of the nervous system and the pathogenesis of neurodegenerative diseases. Ost has been shown to modulate the Notch signaling pathway through the upregulation of miR-9, thereby increasing cell viability and preventing death in neural stem cells. The Notch signaling pathway, which is crucial for regulating cell fate decisions, proliferation, and differentiation in neural tissues, is often dysregulated in conditions such as AD. By increasing the levels of miR-9, which is a target of *HES1* mRNA, Ost appears to exert a protective effect on neural stem cells expressing APP, potentially counteracting the adverse effects of amyloid-related neurodegeneration (Li et al., 2017b). A previous study indicated that the miR-9/hairy1 pathway also regulates cell proliferation by modulating the expression of cyclin D and p27, key regulators of the cell cycle (Bonev et al., 2011). miR-29 regulator of DNA methyltransferase 3A (DNMT3A), an enzyme responsible for DNA methylation, plays a critical role in regulating gene expression (Swahari et al., 2021). By regulating DNMT3A, miR-29c modulates the DNA methylation landscape, influencing the expression of various genes involved in neuronal development. Another key gene regulated by miR-29c is BDNF, a crucial protein that supports neuronal survival, growth, and proliferation (Yang et al., 2015). Through its effect on BDNF expression, miR-29c promotes neuronal proliferation, suggesting its potential as a therapeutic target for enhancing neurogenesis and treating neurodegenerative conditions. By passing through neural progenitors, miR-124 directly reprograms fibroblasts into γ-aminobutyric acid (GABA)-ergic neurons. GABAergic neurons are the main inhibitory neurons in the human brain and have an irreplaceable effect on the pathological mechanism of AD. Deficits in GABAergic neurons can lead to the progression of AD (Gu et al., 2022; Rivera et al., 2023). *PTBP1*, a target gene of miR-124, is notably downregulated in neurons. Makeyev et al. (2007) reported that lowering PTBP1 levels facilitates the proper splicing and accumulation of PTBP2 mRNA and enhances neuronal maturation. These findings suggest a regulatory relationship in which PTBP1 functions as a key factor influencing the splicing process of PTBP2. Moreover, inhibiting PTBP1 alone has been shown to induce fibroblasts to transdifferentiate into functional neurons (Xue et al., 2013). miR-124 promotes proliferation and neural stem cell differentiation into neurons by activating the Wnt/β-catenin signaling pathway and decreasing the expression of disheveled binding antagonist of beta-catenin 1 (DACT1), ultimately promoting neuronal growth (Jiao et al., 2018). Moreover, inhibition of miR-146a expression promotes neurite growth and may be linked to the STAT1/MYC pathway, which regulates key cellular processes (Ma et al., 2021). In summary, miRNAs are pivotal in promoting neuronal development and synaptic integrity, positioning them as promising therapeutic targets in efforts to counteract AD-related neuronal damage and cognitive decline. We demonstrate the targets of miRNAs that regulate neuronal death and development in **Figure 7**.

Furthermore, the expression level of miR-124 dynamically changes in AD, and its activation or repression is regulated by a multilevel upstream regulatory network. It has been shown that decreased expression levels of REST due to factors such as Aβ and neuroinflammation lead to excessive transcription of miR-124, which triggers hyperphosphorylation and aggregation of tau proteins, disrupting the miRNA network and exacerbating the pathological process of AD (Hou et al., 2020). PTBP1 directly inhibits miR-124 expression by binding to pri-miR-124-1 and blocking DROSHA cleavage in the nucleus (Yeom et al., 2018). Notably, the interaction between PTBP1 and miR-124 results in a self-reinforcing cycle, wherein depletion of PTBP1 increases the expression of miR-124, which in turn further inhibits PTBP1 expression, thereby driving the cellular transition to the neuronal state. Further studies revealed that miR-124 expression was also competitively regulated by noncoding RNA networks. The silencing of the long noncoding RNA XIST (lncRNA XIST) and the lncRNA NEAT1 serves as upstream regulatory events by significantly upregulating the expression of miR-124, which subsequently inhibits the transcriptional activity of the downstream target gene BACE1, thereby attenuating Aβ deposition (Zhao et al., 2019; Yue et al., 2020). However, the exploration of noncoding RNA interaction networks in AD remains exploratory, and only a few molecules have been identified to have regulatory relationships with miR-124. However, its prevalence in AD and its synergistic effects with other regulatory elements (e.g., circular RNA) still require systematic verification. Notably, epigenetic modifications have emerged as critical players within these networks, potentially bridging miRNA dynamics and AD pathology.

## Regulation of MicroRNA Biogenesis in Alzheimer’s Disease

### Transcription, splicing, and modification of miRNA

The discovery of RNA base modification in the 1960s led to extensive research into post-transcriptional RNA modifications, which play pivotal roles in RNA function and gene expression regulation (Courtney et al., 2019). In contrast to traditional RNA, miRNA, a subclass of small RNA, also undergoes similar types of modifications (Shi et al., 2022a). These modifications play pivotal roles in regulating miRNA biogenesis and stability as well as the interactions of miRNAs with their target mRNAs. miRNA biogenesis begins with RNA polymerase II- or III-dependent transcription of long pri-miRNAs, which form characteristic hairpin structures. DGCR8 recognizes these pri-miRNAs, recruiting the Drosha enzyme to cleave them into pre-miRNAs. m6A modifications play an important role in the regulation of miRNA biogenesis by influencing the processing of pri-miRNAs. Methyltransferase-like (METTL)3 methylates pri-miRNAs, which facilitates recognition by heterogeneous nuclear ribonucleoprotein A2/B1 (HNRNPA2/B1). This interaction promotes the binding of DGCR8 to pri-miRNAs, accelerating their processing into mature miRNAs (Alarcón et al., 2015).

### A brief overview of modification methods in miRNA

Enzymes that write, read, and erase RNA modifications play critical roles in regulating RNA function (Qu et al., 2022; Satterwhite and Mansfield, 2022). Writers add specific modifications to nucleotides; readers recognize and interpret these modifications; and erasers remove them, enabling dynamic regulation of gene expression. In this regard, m^6^A, 2′-O-methylation (Nm), 5-methylcytosine (m^5^C), and pseudouridine (Ψ) have attracted attention for the modification of miRNA.

### N^6^-methyladenosine modification of miRNA in Alzheimer’s disease

m^6^A is a prominent RNA modification catalyzed by METTL3, METTL14, and METTL16, with fat mass and obesity-associated protein and ALKB homolog 5 acting as demethylases (Schumann et al., 2016; Yi et al., 2020b). m^6^A recognition is facilitated by reader proteins, including members of the YTH domain family (YTHDF1/2/3, and YTHDC1/2), heterogeneous nuclear ribonucleoproteins (HNRNPC and HNRNPA2/B1), translation initiation factors like eIF3, and the insulin-like growth factor 2 mRNA-binding protein (IGF2BP) family (IGF2BP1/2/3) (Huang et al., 2018; Shi et al., 2019). NOP2/Sun RNA methyltransferase family member 2 (NSun2), a brain-enriched RNA methyltransferase, is involved in the methylation of non-coding RNAs, including miRNAs. Reduced NSun2 expression in patients with AD has been shown to lead to a decrease in m^6^A modification of miR-125b, resulting in its enhanced function (Kim et al., 2023). This alteration promotes tau protein phosphorylation, a key pathological feature of AD. These results suggest that altered miRNA modifications are crucial in the pathogenesis of AD. These changes may disrupt key regulatory processes, offering potential implications for therapeutic targeting of miRNA modifications to combat AD. A growing body of evidence has highlighted the critical role of m^6^A modifications in cancer. In particular, m^6^A has been shown to affect various oncogenic pathways by regulating the expression of specific miRNAs, which, in turn, modulate the stability and activity of target genes. Chen et al. (2024a) revealed that the m^6^A modification plays a central role in promoting the tumorigenic phenotype of arsenite-transformed BEAS-2B cells, a cell model used to study lung cancer. Their study showed that METTL3 upregulates miR-106b-5p, an miRNA involved in cancer progression through the METTL3/miR-106b-5p/BNC2 pathway, thereby promoting tumor cell proliferation and migration. YTHDF2, an m^6^A reader, recognizes m^6^A modifications in pre-miR-126 and recruits AGO2, enhancing pre-miR-126 maturation (Zhang et al., 2024d). Zhang et al. (2024d) observed that this process promotes the progression of acute myeloid leukemia. METTL14, an m^6^A methyltransferase, is upregulated in breast cancer tissues, and its overexpression has been found to promote breast cancer cell migration and invasion. METTL14 regulates hsa-miR-146a-5p and enhances cancer cell migration and invasion (Yi et al., 2020a).

Despite the growing recognition of RNA modifications as crucial regulators of gene expression, research on miRNA modifications in neurodegenerative diseases, including AD, remains in the early stages. miRNAs are known to regulate the expression of genes involved in various cellular processes pivotal in maintaining brain function. Changes in miRNA levels or function have been implicated in the pathogenesis of AD, but the specific roles of miRNA modifications in the disease process remain largely unexplored. Since AD is characterized by complex and multifactorial alterations at the molecular level, the exploration of miRNA modifications could yield important findings and open new strategies for diagnosis and treatment. Beyond miRNAs, circular RNAs have gained attention as important regulators in neurodegenerative disorders like AD. Circular RNA regulating synaptic membrane exocytosis 2 (circRIMS2) was found to be significantly upregulated in a mouse model of AD, and this upregulation was regulated by METTL3-dependent m6A modification. Importantly, the overexpression of circRIMS2 in the AD mouse model led to noticeable deficits in synaptic function and memory, two key features of AD pathology (Wang et al., 2023d). This study expanded the existing understanding of RNA modifications in AD by highlighting the role of circular RNA modifications.

### Small RNA modifications hold great promise in disease monitoring and prognosis

miRNAs have been found to play several roles in biogenesis regulation, including prevention, prediction, and prognostic monitoring. In a study involving 57 cases of human neural tube defects, researchers found an association between aberrant methylation of HS-let-7g and disturbances in folate metabolism, suggesting that folate deficiency during pregnancy may contribute to neural tube defects (Wang et al., 2017a). These results highlight the potential for improved nutrition in early pregnancy, particularly folate supplementation, to prevent such defects. Yan et al. (2013) identified significant differential expression of small RNA modifications in the liver of diabetic mice. These changes in small RNA profiles may play a role in the pathophysiology of diabetes and could serve as potential biomarkers for therapeutic strategies aimed at improving liver function and metabolism in diabetic conditions. In the context of cancer, Konno et al. (2019) analyzed miR-17-5p methylation levels in serum samples and found that these levels could distinguish patients with early-stage pancreatic cancer from healthy controls with high sensitivity and specificity. The methylation of miR-17-5p, an miRNA known to be involved in regulating cell proliferation and apoptosis, was significantly altered in the serum of patients with pancreatic cancer in comparison with that in healthy individuals. The ability to detect early-stage pancreatic cancer through noninvasive serum testing holds promise for improving diagnosis and patient outcomes. Zhang et al. (2020) showed that small RNA modifications were significantly altered in AD samples in comparison with normal controls. These changes could represent a novel signal associated with the pathogenesis and progression of AD. Although the exploration of small RNA modifications in neurodegenerative diseases, particularly AD, is in the nascent phase, the findings of early studies indicate that this approach holds promise. Although the existing research has largely focused on genetic and protein-based mechanisms, the growing recognition of the role of small RNAs, such as miRNA itself, offers a new dimension to investigations of AD pathology. As funding and collaborative efforts increase, substantial breakthroughs will soon emerge, offering hope for more effective treatments in the future.

## Diagnostic Potential of MicroRNAs in Alzheimer’s Disease

The main imaging features of AD include atrophy of the hippocampus, internal olfactory cortex, and amygdala. Blood flow and metabolism in the hippocampal region can be visualized by fludeoxyglucose F18 (F-FDG)-PET imaging, and Aβ-PET and tau-PET can enable early visualization of disease-specific protein accumulations. However, these imaging methods are costly, and large-scale screening using these methods is challenging. Since AD diagnosis cannot be fully understood merely through assessment of two proteins, additional diagnostic biomarkers are urgently required.

### miRNAs in cerebrospinal fluid

Kiko et al. (2014) measured six candidate miRNAs in the cerebrospinal fluid (CSF) of AD patients and normal subjects. The results revealed that CSF levels of miR-34a, miR-125b, and miR-146a were significantly lower in AD patients than in the controls. Conversely, the levels of miR-29a and miR-29b in the CSF were significantly greater in AD patients than in the controls, with their altered expression in the CSF potentially reflecting the molecular changes associated with the disease. Furthermore, in a study measuring 754 miRNAs in CSF via the Megaplex TaqMan protocol, five miRNAs were differentially expressed in AD patients, and seven miRNAs were differentially expressed in late-onset AD patients after stratification by age (van Harten et al., 2015). Lusardi et al. (2017) analyzed miRNAs in the CSF of 50 AD patients and 49 controls, identifying 36 miRNAs that differentiated between AD and control CSF and improving the diagnostic performance of the model with the addition of the *ApoE* genotype. In summary, CSF biomarkers have long been valuable tools for diagnosing and monitoring neurological diseases.

### miRNAs in blood

In clinical applications, CSF biomarkers are often limited by the invasive procedure of lumbar puncture, which is uncomfortable for patients and carries some risk of complications (Reis et al., 2024). As a result, interest in the development of alternative blood-based biomarkers has increased (Janelidze et al., 2020; Karikari et al., 2021). Blood sampling is noninvasive, easier to perform, and generally more acceptable to patients, making it a more viable option for large-scale screenings and repeated testing. Next-generation sequencing of miRNAs in blood samples from 48 AD patients and 22 controls identified 12-miRNA profiles capable of distinguishing AD patients from controls with high accuracy, specificity, and sensitivity (Leidinger et al., 2013). Satoh et al. (2015) analyzed miRNA expression in blood samples from 48 AD patients and 22 healthy controls and identified 27 miRNAs that were differentially expressed between the two groups. Another study of whole blood samples revealed that diminished levels of miR-9-5p, miR-106a-5p, miR-106b-5p, and miR-107 were linked to an increased risk of AD (Yılmaz et al., 2016).

#### miRNAs in plasma

Using capture technology, Piscopo et al. (2023) demonstrated that miR-92a-3p and miR-320a are reliable biomarkers for distinguishing AD patients from control subjects in plasma. Through miRNA profiling in patient plasma, Kenny et al. (2019) revealed that elevated miR-206 levels predict cognitive decline in AD patients. Analysis of plasma and brain miRNA levels in two cohorts, including controls and patients with mild cognitive impairment (MCI), AD, and frontotemporal dementia, revealed that miR-92a-3p, miR-181c-5p, and miR-210-3p form a molecular signature that may serve as a potential biomarker for AD (Siedlecki-Wullich et al., 2019). Analysis of plasma miRNAs in a cohort of symptomatic AD patients, healthy controls, and preclinical AD patients Cosín-Tomás et al. (2017) identified miR-34a-5p and miR-545-3p as promising early biomarker candidates for AD. Quantitative reverse transcription polymerase chain reaction analysis revealed significantly lower plasma levels of miR-34a and miR-146a in AD patients than in controls, and an increased abundance of miR-34c in circulating plasma has been identified in AD patients (Bhatnagar et al., 2014; Kiko et al., 2014). The analysis demonstrated that miR-103 and miR-107 effectively differentiated AD patients from healthy controls, and miR-1185-2-3p, miR-1909-3p, miR-22-5p, and miR-134-3p have been implicated in the diagnosis of aMCI, a precursor to AD (Wang et al., 2020; He et al., 2021).

#### miRNAs in serum

The downregulation of miR-125b in the serum of AD patients was identified by Tan et al. (2014b), demonstrating its high specificity and sensitivity. A serum miRNA panel consisting of miR-98-5p, miR-885-5p, miR-483-3p, miR-342-3p, miR-191-5p, and miR-let-7d-5p was identified through genome-wide miRNA expression analysis as effective in discriminating AD patients from healthy controls (Tan et al., 2014a). Additionally, significantly lower serum levels of miR-125b, miR-23a, and miR-26b were confirmed in AD patients through the measurement of 84 miRNAs (Galimberti et al., 2014). Using Solexa sequencing, two studies reported that four miRNAs (miR-31, miR-93, miR-143, and miR-146a) were significantly reduced in the sera of AD patients (Dong et al., 2015; Kumar et al., 2017). Other miRNAs, including miR-137, miR-181c, miR-9, miR-29a, miR-29b, miRNA-210, miR-223, miR-29c-3p, miR-19b-3p, miR-133b, miR-28-3p, miR-331-3p, miR-128 and miR-106b, have been found to be dysregulated in the serum of AD patients compared with healthy controls, suggesting their potential as biomarkers for predicting AD (Jia and Liu, 2016; Wu et al., 2017; Yang et al., 2019; Zhao et al., 2020; Liu and Lei, 2021; Zhang et al., 2021b; Madadi et al., 2022).

### miRNAs in exosomes from cerebrospinal fluid and blood

Exosomes, which are secreted by nearly all mammalian cells, mediate cellular communication and can alter the behavior of neighboring or distant cells through paracrine or endocrine signaling (Yang et al., 2020a). Exosomal miRNAs remain stable in body fluids, and as natural carriers of miRNAs, exosomes can cross the blood‒brain barrier. Changes in miRNA expression in exosomes from CSF or peripheral blood could serve as potential biomarkers for diagnosing AD. Compared with those of healthy controls, the miRNA expression profiles of exosomes isolated from the CSF of patients with biomarker-confirmed AD revealed differential expression of miR-16-5p, miR-125b-5p, miR-451a, and miR-605-5p (McKeever et al., 2018). In a study of 760 miRNAs in the CSF of 10 AD patients and 10 healthy subjects, miR-9-5p, miR-134, and miR-598 were selected for further analysis, with miR-9-5p and miR-598 emerging as potential biomarkers for AD (Riancho et al., 2017).

Next, we discuss the diagnostic value of miRNAs in exosomes in the blood. Next-generation sequencing analysis of plasma exosomal miRNAs revealed eight differentially expressed miRNAs between AD patients and normal controls. Three miRNAs—miR-423-5p, miR-369-5p, and miR-23a-3p—were significantly elevated in AD, whereas five miRNAs—miR-204-5p, miR-125a-5p, miR-1468-5p, miR-375, and let-7e-5p—were significantly decreased in AD samples (Nie et al., 2020). Using Illumina deep sequencing, Lugli et al. (2015) compared samples from 35 people with a clinical diagnosis of AD dementia to 35 age- and sex-matched controls and identified 20 miRNAs (miR-23b-3p, miR-24-3p, miR-29b-3p, miR-125b-5p, miR-138-5p, miR-139-5p, miR-141-3p, miR-150-5p, miR-152-3p, miR-185-5p, miR-338-3p, miR-342-3p, miR-342-5p, miR-548at-5p, miR-659-5p, miR-3065-5p, miR-3613-3p, miR-3916, and miR-4772-3p, and miR-5001-3p) that showed significant differences in the AD group in the initial screen. miR-212 and miR-132 were found to be downregulated in neural-derived plasma exosomes from AD patients, suggesting their potential as biomarkers for AD (Cha et al., 2019). Cheng et al. (2015) analyzed miRNAs in serum exosomes from AD patients and identified 16 miRNAs, which were consistent with the findings of neuropsychological and neuroimaging assessments associated with AD. In AD patients, serum exosomes showed upregulation of miR-135a and miR-384 and downregulation of miR-193b compared with those in normal controls. Receiver operating characteristic (ROC) analysis revealed that combinations of miR-135a, miR-193b, and miR-384 outperformed other combinations for early AD diagnosis (Yang et al., 2018b). In another study, Liu et al. (2021) identified miR-135a in ATP-binding cassette transporter A1 (ABCA1)-labeled exosomes as a potential serum biomarker for AD, particularly in the early stages. Additionally, miR-30b-5p, miR-22-3p, and miR-378a-3p were found to be significantly dysregulated in the serum exosomes of AD patients (Dong et al., 2021). The combination of these three miRNAs demonstrated enhanced diagnostic ability for AD, offering a promising avenue for early detection and potentially improving the accuracy of AD diagnosis through noninvasive biomarkers.

These results add to the growing body of evidence supporting the role of specific miRNAs in neurodegenerative diseases and open possibilities for the use of miRNAs as diagnostic or prognostic tools in AD. However, the results thus far clearly show some differences. We speculate that the reason for these inconsistent findings, in addition to different assay techniques and experimental models, may be differences in the timing of sample collection. In the early stages of AD, minor pathologic mechanisms may not significantly affect the homeostasis and balance of patients; thus, miRNAs may not be significantly altered or may even be upregulated to combat neuroinflammation. Recently, using small RNA sequencing to analyze plasma miRNAs at different clinical stages of AD, Krüger et al. (2024) reported that miRNA markers not only facilitated AD diagnosis but also exhibited varying expression patterns corresponding to the temporal progression of AD. These results suggest that the time-dependent expression of miRNAs reflects the stages of AD pathology. In any case, more in-depth research and accurate differentiation are needed to advance the field.

### Current clinical trials of miRNAs for the diagnosis of Alzheimer’s disease

In this section, we provide a comprehensive overview of the current clinical trials investigating the use of miRNAs for the diagnosis of AD. This section aims to offer a thorough perspective on the ongoing efforts exploring miRNAs as diagnostic biomarkers for AD, considering their potential to reflect disease-specific molecular signatures. We review the design, scope, and outcomes of these trials, shedding light on the progress made so far and identifying the challenges that need to be addressed to integrate miRNA-based diagnostics into clinical practice for AD. Amnestic mild cognitive impairment (aMCI), a prodromal stage of AD, is characterized by noticeable memory problems that are greater than expected for the person’s age but not severe enough to meet the criteria for dementia. Identifying biomarkers that can detect aMCI early is crucial for timely intervention and potential therapeutic strategies. Wang et al. (2015) examined the relationship between plasma miRNA-107 expression and *BACE1* mRNA in aMCI. Their findings demonstrated that miRNA-107 levels can effectively distinguish between patients with aMCI and healthy controls, highlighting the potential of miRNA-107 as a diagnostic biomarker for early AD. In a study of 20 patients with aMCI and 24 cognitively normal controls, T1-weighted magnetic resonance imaging (MRI) scans were used to assess cortical measures, including cortical thickness, surface area, and localized gyrus index (Wang et al., 2016c). The findings revealed significant structural alterations in key left-brain regions associated with memory, language, and emotion in the patients with aMCI. These changes were strongly correlated with plasma miRNA-107 levels and *BACE1* mRNA expression, highlighting a potential link between brain morphology and molecular biomarkers in aMCI. Furthermore, ongoing clinical trials, such as NCT02045056 and NCT04840823, are exploring the safety and efficacy of miRNAs in combination with drugs and external stimuli for the prevention and treatment of AD. A randomized, double-blind, placebo-controlled prospective clinical study (NCT02045056) conducted by the University of Kentucky in 2019 recruited participants who were randomly assigned to receive treatment with gemfibrozil (600 mg orally twice a day) or placebo for 52 weeks (NCT02045056). This study aimed to assess the safety and efficacy of gemfibrozil in modulating miR-107 levels for the prevention of AD in individuals with intact cognition and patients with mild cognitive impairment (MCI). The authors found that gemfibrozil improved miR-107 levels in patients with MCI, reduced Aβ deposition, and restored memory and learning abilities. These preventive trials provide a broader field of exploration for miRNA-based therapies, paving the way for future applications in neurodegenerative disease treatment.

The development of miRNA-based diagnostics for neurodegenerative diseases has shown considerable promise, but translating these findings into clinical practice remains challenging. Although several clinical trials investigating miRNAs as diagnostic biomarkers for neurodegenerative diseases are ongoing (NCT04961450, NCT04509271, NCT02859428, and NCT05243017), many have not yet yielded results. A prospective cohort clinical study conducted by the Peking University Third Hospital in China spanning a period of 10 years (NCT04961450) aims to focus on biomarkers associated with the spectrum of motor neuron diseases and frontotemporal dementia. This study will include adults diagnosed with motor neuron disease/frontotemporal dementia and healthy controls. Clinical samples, including blood, saliva, feces, muscle, and nerve tissue, will be collected from both groups to extract specific proteins, miRNA, and DNA. Finally, biostatistical analyses will be performed to investigate biomarkers and potential pathogenesis related to the motor neuron disease/frontotemporal dementia spectrum of diseases. A 1-year case-control clinical study evaluating the diagnostic accuracy of biomarkers for MCI was conducted at the Shanghai Mental Health Center (NCT04509271). It aimed to study the reliability and validity of plasma miRNAs for early diagnosis of MCI due to AD. The study collected peripheral plasma and non-coding RNA from individuals diagnosed with MCI. miRNA assay kits were used for diagnostic analysis, thus enhancing early clinical identification of patients with MCI. Factors such as limited participant numbers, invasive testing methods, and challenges in data comparison lead to the bottleneck in the research of miRNA biomarkers for AD diagnosis underscoring the need for further validation and refinement of miRNA-based diagnostic approaches. Additionally, some diagnostic techniques for assessing miRNA expression levels, such as those using cerebrospinal fluid, can be invasive and difficult for participants to accept. The variability in miRNA expression across different stages of disease and among individual patients further complicates efforts to establish reliable diagnostic biomarkers. Despite these challenges, numerous trials continue to explore the role of miRNAs as potential biomarkers for AD. As these trials progress, they may eventually provide the necessary evidence to integrate miRNAs into clinical diagnostics and therapies for neurodegenerative diseases.

## Therapeutic Potential of MicroRNAs in Alzheimer’s Disease

The existing therapeutic agents for AD, including common cholinesterase inhibitors and N-methyl-d-aspartate antagonists, primarily focus on slowing disease progression and cannot reverse disease progression. Moreover, these drugs often have multiple adverse effects, including renal and gastrointestinal impairment. Alternative therapies like music therapy or exercises can only be used as adjunct modalities for early-stage patients (Rossi et al., 2024). These limitations indicate an urgent need to develop innovative therapies, and miRNAs have shown promise in this regard.

### Clinical application potential of miRNAs in Alzheimer’s disease

Therapeutic strategies targeting miRNAs primarily involve the application of miRNA mimics or inhibitors. Numerous preclinical studies have been performed using various animal models to evaluate the potential therapeutic benefits of miRNAs in various diseases, and analogs of miR-124 have been shown to ameliorate many of the pathologies of AD in a variety of animal models. Li et al. (2019a) revealed that lentivirus-mediated upregulation of miR-124 improves cerebral microvascular function by regulating C1ql3. Similarly, Zhou et al. (2019) employed an adeno-associated virus (AAV) to deliver miR-124-3p into APP/PS1 mice, resulting in a significant reduction in Aβ plaque deposition. Neurons can release exosomes containing miR-124 into microglia to influence microglial polarization and improve neuroinflammation in AD, which represents a novel therapeutic strategy (Garcia et al., 2022). In addition, syringin, a plant extract, has been shown to enhance miR-124-3p expression by reducing BID expression, thus preventing Aβ-induced neurotoxicity (Zhang et al., 2021c). In another study, the use of astaxanthin to treat AD-like rats led to a substantial increase in miR-124 expression, bringing miR-124 levels in the cortex and hippocampus close to normal, suppressing the pathological features of AD such as BACE1 expression and microglial activation, and enhancing the antioxidant pathway of Nrf2, which highlights the critical role of miR-124 (Hafez et al., 2021). In AD mouse models, miR-331-3p and miR-9-5p antagonists enhanced Aβ clearance and improved cognition and mobility (Chen et al., 2021). These findings suggest that targeting these miRNAs may be a therapeutic strategy for mitigating AD-related pathology and improving neurological function in later stages of the disease. Jahangard et al. (2020) successfully packaged miR-29b into exosomes and administered these exosomes to an AD rat model; their findings demonstrated that exosomal delivery of miR-29b successfully upregulated miR-29b expression in the hippocampus and downregulated the expression of its target genes, NAV3 and BIM. This resulted in improved cognitive function, providing a novel strategy for AD treatment through exosome-based miRNA delivery. Wu et al. (2025) established a solid foundation for considering miR-29c as a viable therapeutic target by injecting miR-29c-3p agomir into an AD model. This intervention led to a significant decrease in the expression of Lamr1-ps1 and BACE1, both of which are involved in Aβ production and deposition. Consequently, Aβ deposition was reduced, which, in turn, contributed to improvements in spatial learning and memory deficits.

The number of miRNA-based therapeutics in clinical trials has been steadily increasing, reflecting the growing interest and potential of miRNAs as therapeutic targets. As research continues to advance, several miRNA-based drugs are now awaiting regulatory approval, signaling a promising future for the use of miRNA-based therapies in clinical practice. However, most of these drugs are cancer-specific. For instance, LNA-i-miR-221, a 13-mer locked nucleic acid (LNA) inhibitor in phase I trials, has demonstrated favorable safety and efficacy by downregulating miR-221 and upregulating its targets (cyclin-dependent kinase inhibitor 1B [CDKN1B] and PTEN), showing significant antitumor activity (Tassone et al., 2023). This approach holds promise for treating solid tumors, especially in advanced, treatment-resistant cancer cases. Regulus Therapeutics Inc. developed RGLS5579, an miRNA-based drug aimed at targeting and inhibiting miR-10b. As a clinical candidate for glioblastoma multiforme, a highly aggressive and treatment-resistant cancer, RGLS5579 is currently undergoing active clinical trials (Ghosh et al., 2018). Because of its ability to inhibit miR-10b, this drug holds substantial potential in the treatment of glioblastoma multiforme. The ongoing trials have showcased the promise of miRNA-targeting strategies in cancer therapy, particularly for tumors like glioblastoma multiforme that are difficult to treat with conventional therapies. RGLS5579 represents a novel approach in cancer treatment, highlighting the therapeutic possibilities of miRNA modulation in oncology. ALS is a progressive neurodegenerative disorder characterized by the loss of motor neurons, which leads to muscle weakness, paralysis, and, ultimately, respiratory failure. Despite the major advancements in the understanding of the pathogenesis of ALS, therapeutic options remain limited. miR-155 has been identified as a key contributor to the inflammatory responses in ALS, and its inhibition could potentially slow the progression of neuronal damage. The use of mRG-107 to specifically target miR-155 represents a novel therapeutic strategy aimed at addressing these mechanisms. The progression of mRG-107 into preclinical trials marks a crucial step in the development of targeted treatments for ALS, which has traditionally been a neglected area of research. AD, which is characterized by progressive cognitive decline and neurodegeneration, remains a major challenge in both clinical and research settings. The role of miRNAs such as miR-107 in modulating disease-related pathways offers a novel avenue for the treatment of AD. miR-107 has been shown to influence Aβ processing and reduce inflammation, which are two major contributors to AD pathogenesis. The use of cross-sectional case observations allowed researchers to analyze the immediate effects of miR-107 in clinical settings, comparing cognitive function and biomarkers before and after treatment (NCT01819545). This methodology can help identify not only the potential efficacy of miR-107 but also its safety profile in the context of AD. A 3-month exercise intervention program was designed to explore the potential effects of myokines and miRNAs on individuals showing MCI associated with AD and Parkinson disease (NCT02253732). The program specifically focused on understanding how these biological molecules interact with the neurodegenerative processes underlying both MCI in AD and motor symptoms in PD. By promoting the release of beneficial myokines and modulating miRNA profiles, the exercise intervention could offer a dual therapeutic effect, potentially improving cognitive and motor functions in these patients.

The existing data also highlight the substantial challenges in the clinical application of miRNAs for brain disorders. These obstacles are evident in the limited number of miRNA-based clinical trials focused on neurodegenerative diseases, especially AD. Despite the promising therapeutic potential of miRNAs in modulating disease-related pathways, several factors hinder their clinical translation, including issues related to delivery mechanisms, specificity, and long-term stability. Furthermore, the complexity of neurodegenerative diseases, which are characterized by multifaceted pathophysiological processes, poses an additional barrier to the successful implementation of miRNA-based therapies. Consequently, a substantial gap has persisted between preclinical research and clinical application in the context of miRNA therapeutics for AD and other brain disorders.

### miRNAs modulate direct reprogramming to treat Alzheimer’s disease

Compared with traditional clinical treatments, cellular reprogramming technology provides a new perspective on AD treatments. Since Takahashi and Yamanaka (2006) developed induced pluripotent stem cell (iPSC) technology, which can reprogram mouse fibroblasts to a pluripotent state by applying retroviruses to deliver four transcription factors, including Octamer-binding transcription factor 3/4 (Oct3/4), SRY-related HMG-box transcription factor 2 (Sox2), the cellular myelocytomatosis oncogene c-Myc and Klf4, the field of cellular reprogramming has undergone rapid development. This type of therapy differs from embryonic stem cell technology in that it uses the patient’s own somatic cells to differentiate into neuronal cells, blood cells, and primordial germ cells, which avoids immunological rejection and the ethical issues associated with the disruption of the embryo (Volarevic et al., 2018). Currently, this technology is applied to model the pathogenesis and preclinical treatment of AD. For example, astrocytes formed by iPSC technology in the AD environment recapitulate the abnormal morphology and dysfunction of neuronal networks, and embryonic stem cell-derived protein-iPSCs transplanted into an AD mouse model ameliorate cognitive dysfunction and reduce Aβ plaque deposition (Cha et al., 2017; Jones et al., 2017). However, cells programmed via the iPSC technique have a pluripotent state that may lead to tumor formation; thus, a better approach involving direct reprogramming may have greater potential (Chambers and Studer, 2011). Since Volarevic et al. (2018) first successfully achieved *in vitro* direct reprogramming of mouse and human fibroblasts into neurons, miR-124 has emerged as a key factor in this process. Studies have demonstrated that miR-9/9* and miR-124 can downregulate ubiquitin-specific protease 14 (USP14) and induce repression of the enhancer of Zeste homolog 2-RE-1 silencing transcription factor (EZH2-REST) axis to promote direct reprogramming of human fibroblasts into neurons (Drouin-Ouellet et al., 2017; Lee et al., 2018). Moreover, Lu et al. (2021) reported that the synergy between miR-124 and embryonic lethal abnormal vision drosophila-like 3 (ELAVL3), an RNA-binding protein, enhances reprogramming and promotes neuronal maturation. In mouse models, Guo et al. (2023) and Papadimitriou et al. (2023) reported that miR-124 inhibits Notch1 and DLL4 while binding to NeuroD1 and promotes the direct reprogramming of astrocytes to neurons *in vivo*, and Papadimitriou et al. (2023) reported that miR-124 inhibits the RNA-binding protein Zfp36L1 in the neurogenic interactome and promotes the reprogramming of astrocytes into neurons. Notably, reprogramming astrocytes into neurons *in vivo* does not cause immunosuppression, and the large astrocyte population provides the basis for the feasibility of this technique. However, the clinical use of such methods in humans is still in its infancy, and the neurons formed by reprogramming can still be degraded by the environment in the brains of AD patients; as a result, regenerative medicine has a long way to go in treating brain disorders (Gascón et al., 2017; Sun et al., 2024).

### miRNA delivery strategies for disease treatment

As discussed above, miRNAs have notable therapeutic potential for CNS disorders, especially AD. However, safe transport of miRNAs across the blood-brain barrier to the CNS remains a challenge. In this section, we have categorized the existing delivery strategies into non-viral vectors and viral vectors, offering insights into their relative benefits and limitations for AD-targeted therapies.

#### Non-viral vector nanoparticles

Unlike other larger-scale materials, nanoparticles offer unlimited possibilities in the biomedical field due to their small size, safety, stability, and other favorable characteristics. Since the late 20^th^ century, nanotechnology has made rapid progress, and nanoparticles have been employed in a variety of functions in the biomedical field (Jain et al., 2024). Nanocarriers are currently used for miRNA delivery, release, and therapy. These vectors can increase the stability of miRNAs, protecting them from degradation and increasing target accumulation (Ashrafizadeh et al., 2023). However, nanoparticles have certain limitations, including those related to size, shape, and surface charge; these limitations are also closely related to the delivery capacity and post-delivery effects (Gong et al., 2023). Therefore, choosing the right nanomaterials is critical. Because liposomes show low toxicity and can be easily fabricated and modified, the use of liposomes to deliver miRNAs is one of the most widely studied non-viral vector methods. Liposomal nanoparticles can also stabilize nucleic acids to prevent miRNAs from being degraded, thereby increasing efficiency (Zhang et al., 2025a). Hsu et al. (2013) demonstrated the efficacy of LNP-DP1 in delivering miR-122 to hepatocellular carcinoma cells, resulting in the normalization of hepatic gene expression. This targeted approach offers a potential therapeutic strategy for hepatocellular carcinoma, although further investigation into long-term effects and optimization of delivery parameters are necessary. Through liposomal delivery of anti-miR-124 to the vortex worm (*Platynereis dumerilii*), Sasidharan et al. (2017) elucidated the regulatory influence of miR-124 on regeneration. Their findings highlight the importance of miR-124 in these regenerative processes. Other nanomaterials, including polymeric nanomaterials and inorganic nanoparticles, have also shown excellent results. Polylactic-co-glycolic acid, which is produced by random polymerization of lactic acid and hydroxy acetic acid, has shown non-toxic and biodegradable properties with excellent biocompatibility (Lee et al., 2019). One study utilized polylactic-co-glycolic acid to deliver anti-miR-141-3p and improve ischemic stroke in a mouse model (Dhuri et al., 2021), and another study showed the therapeutic efficacy of a ketoprofen and miR-124 co-loaded polylactic-co-glycolic acid microsphere delivery strategy in a rat model of rheumatoid arthritis (Yu et al., 2018). The inherent stability and biocompatibility of inorganic nanoparticles make them attractive candidates for targeted miRNA delivery systems. The scope for precise size control and surface-modification strategies further enhance the efficacy of these systems in controlled miRNA release applications (Wang, 2024). However, Saraiva et al. (2018) showed that while nanoparticles loaded with miR-124 promoted neural stem cell survival and neuronal differentiation *in vitro*, they had no effect in the human stroke environment. Thus, the bioavailability and targeting effects of nanocarriers in the human body require improvement. In addition, the complex mechanisms of the internal environment in the human body may hinder the optimal functioning of man-made nanoparticles in the body, while overly complex nanoparticles engineered to adapt to these complex mechanisms may be difficult to produce in large quantities.

Exosome-associated delivery strategies may address some of these limitations. Exosomes, which are natural nanoparticles with extracellular vesicles of 40–100 nm in size, can cross the blood-brain barrier and carry a variety of cargoes, including proteins, lipids, and miRNAs. In an oxygen-glucose deprivation/reoxygenation-induced mouse model, miR-124-3p was shown to ameliorate HT22 cell injury by piggybacking on exosomes from M2-type microglia (Xie et al., 2023). Similarly, using exosomes from M2 microglia, Zhu et al. (2024) found that transferring miR-124-3p into HT22 cells could protect the cells from glutamate-induced neuronal damage, indicating that the role of exosomes may change depending on the *in vivo* environment. Kong et al. (2024) investigated the influence of miR-124-3p delivered by hippocampal microglial exosomes on cognitive impairment in a mouse model. Using the Morris water maze test, they demonstrated that miR-124-3p shifted the inflammatory balance, suppressing pro-inflammatory and promoting anti-inflammatory signaling and alleviating symptoms of cognitive impairment in the hippocampus. In AD models, Garcia et al. (2022) found that exosomes derived from SWE cells co-cultured with microglia increased miR-124 levels in microglia, demonstrating that miR-124 is transferred from neurons to microglia via an exosome form. These findings may indicate the effectiveness of a new primary treatment approach based on exosome loading of miRNAs, particularly miR-124, in AD. Some recent studies have also achieved breakthroughs in exosome delivery technology. Researchers have innovatively combined Fe65 protein-engineered exosomes with the natural autophagy inducer Cory-B, demonstrating synergistic therapeutic effects in AD treatment through a dual mechanism of targeted delivery and enhanced autophagy (Iyaswamy et al., 2023). Another team successfully developed a novel light-controlled exosome delivery system, mMaple3-mediated protein loading into and release from exosome (MAPLEX), which enabled Clustered Regularly Interspaced Short Palindromic Repeats-associated protein 9 (CRISPR-Cas9)-mediated epigenome editing through a light-controlled protein release mechanism in an AD mouse model (Han et al., 2024). This system successfully inhibited the expression of BACE1 and significantly improved cognitive function in model animals. Notably, miRNAs may synergize with the MAPLEX system-mediated epigenome editing by regulating the gene expression network, thereby enhancing the therapeutic effect. Additionally, the integration of artificial intelligence with extracellular vesicle research is still in its early stages. However, the potential of this approach to guide precise drug delivery through the analysis of large-scale molecular databases should not be overlooked (Greenberg et al., 2023). Nevertheless, since exosomes are closely related to the complex internal environment of the body, much research is required to complete the transition from animal models to use in humans.

#### Viral vectors

AAV, which consists of an **~**26-nm-diameter unenveloped icosahedral viral capsid that encapsulates a linear single-stranded DNA genome of **~**4.7 kb, belongs to the class of viral vectors (Dhungel et al., 2024). Other viral vectors include lentiviruses, which are more suitable for *in vitro* therapy although their loading capacity is twice as high as that of AAV, and adenoviral vectors, which show high cytotoxicity and immunogenicity, despite saving expression time. Thus, AAV, a safe and efficient vector, is considered a promising therapeutic agent, especially in the CNS (Kang et al., 2023; Matsunaga and Gotoh, 2023). For example, Zhou et al. (2019) found that an AAV expressing miR-124-3p significantly reduced Aβ deposition in the brain of APP/PS1 mice and improved dementia performance in AD mice. In other CNS diseases, such as ischemic stroke (Guo et al., 2023), injecting miR-124-enriched AAV into astrocytes of the cerebral motor cortex of rats significantly improved the neurological deficits occurring after ischemic stroke. In conclusion, the utilization of AAV with miR-124 for the treatment of CNS disorders, including AD, has shown considerable promise in various preclinical studies. However, the drawbacks of viral vectors include their low loading capacity and the potential for adverse events due to immunogenicity, both of which highlight the need for continued optimization of design and quality control for AAV. In addition, considering the differences between primate and rodent models, more appropriate preclinical models have to be selected for studies.

## Limitations and Challenges Associated With the Use of MicroRNAs in the Diagnosis and Treatment of Alzheimer’s Disease

Although investigations into the potential of miRNAs as biomarkers have yielded some promising initial results, several major challenges and limitations persist. One of the primary obstacles is the vast number of non-coding genes present in the human genome, which includes a large variety of miRNAs. Despite the progress in understanding the roles of miRNAs, their potential remains underexplored, and many aspects of their function are still poorly understood. Furthermore, beyond miRNAs, other complex non-coding genes, such as long non-coding RNAs and circular RNAs, have not been fully examined, further complicating the task of identifying reliable biomarkers. Furthermore, the diagnostic specificity of certain miRNAs, such as miR-122, is limited. Elevated levels of miR-122 in plasma or serum may indicate liver diseases like hepatitis B virus infection, cirrhosis, or general liver injury, in addition to hepatocellular carcinoma, complicating the use of miR-122 as a biomarker. Standardization and optimization of biomarker-detection methods is crucial to improve miRNA-based technologies. Harmonizing techniques will improve their reliability, consistency, and accuracy in clinical applications. More importantly, multiple large-cohort studies are essential to validate the viability of miRNAs as biomarkers, ensuring their accuracy, sensitivity, and applicability across diverse populations and disease stages.

The potential of miRNA therapy is indeed promising, offering the possibility of precisely targeting disease pathways at the genetic level. However, several limitations and concerns remain to be addressed. One of the primary issues is the potential for adverse reactions associated with miRNA-based therapies. Since miRNAs can regulate multiple targets simultaneously, their modulation could lead to unintended interactions among various cellular pathways. Furthermore, because of the complexity of gene networks, even slight alterations in miRNA expression could trigger a cascade of unintended effects, which may manifest as serious side effects. The MRX34 clinical trial (NCT01829971), which tested a synthetic miR-34a mimic for treating tumors, was prematurely discontinued due to severe immune-related side effects, resulting in the deaths of four patients (Hong et al., 2020). Minimizing the effects on non-target cells is essential to effectively target cells. Choosing the appropriate route of administration and delivery carriers can enhance miRNA specificity and reduce off-target impacts. In this respect, the safety of the carrier material and its compatibility with miRNA are crucial factors in miRNA therapy. The carrier must be biocompatible and non-toxic and must ensure efficient delivery without compromising miRNA integrity (van Zandwijk et al., 2017; Monahan et al., 2021). In addition, determining the optimal dosage and controlling the dosage of miRNA therapy remains a major unresolved challenge. Unlike conventional drugs, where dosages are often based on a standardized range, dosing in miRNA therapies is more complex due to factors such as tissue-specific delivery, miRNA stability, and the potential for off-target effects, potentially leading to situations where therapeutic efficacy remains low despite administration of high doses of miRNA. This can occur if the miRNA is not efficiently taken up by the target cells or if it induces unintended interactions with non-target cells. Thus, fine-tuning the dosage to achieve an effective therapeutic concentration while minimizing adverse effects is essential for the success of miRNA-based therapies. This requires careful dose-response studies, personalized approaches, and the development of monitoring techniques to optimize treatment regimens.

## Limitations

Although we have systematically summarized the current regulatory mechanisms, potential applications, and therapeutic targets of miRNAs in AD, our approach had several limitations: First, the cited literature may have been limited by language barriers (exclusion of non-English studies) and database coverage (lack of searches in preprint platforms), potentially causing omission of key findings. Second, the complexity of AD pathology, in relation to those of Parkinson disease, Huntington disease, and other neurodegenerative disorders, remains unresolved. Additionally, patients with AD often have metabolic diseases, but the interaction mechanisms of miRNAs between AD and metabolic conditions remain underexplored, limiting the ability to distinguish between “causal” and “concomitant” contributions to the pathology. This may be due to the scarcity of relevant studies, and future research is expected to address these questions.

## Conclusions and Outlook

We have summarized the roles of various miRNAs in the different mechanisms of AD, and mainly focused on four representative miRNAs to further explore their relationship with AD. The multifaceted role of miRNAs in AD is outlined in detail in **Additional Table 2**. Our review discusses various pathological mechanisms of AD, including Aβ clearance, tau pathology, neuroinflammation, synaptic dysfunction, and neuronal survival, and death. miRNAs have a broad and complex spectrum of targets relevant to AD, and their biogenesis, processing, and post-transcriptional modifications also play pivotal roles in regulating disease progression. Furthermore, various miRNA delivery strategies have been proposed to enhance the clinical applicability of miRNA-based research. We also considered the potential of miRNA reprogramming as a therapeutic strategy for AD, highlighting its capacity for broad regulatory effects and thereby positioning miRNAs as promising candidates for novel AD treatments.

**Additional Table 2:** Roles of primary miRNAs in Alzheimer's disease

miRNA	Model	Target	Mechanism	Outcome	Reference
**Aβ clearance**					
miR-9	APPswe/PS1dE9 mice	Optineurin	miR-9-5p/Optineurin	Disrupt autophagic processes and potentially accelerate neurodegeneration	Chen et al., 2021
miR-29	SPF C57BL/6J mice; PC12 cells; Sprague- Dawley rats	BACE1	BACE1/APP	Reduce the accumulation of Aβ and attenuate neurotoxicity	Cao et al., 2021b; Li et al., 2021a; Sha et al., 2021; Wang et al., 2023c; Wu et al., 2025
	β-CTF/C6 cell; 3xTg-AD mouse model	PSEN1	PSEN1/γ-secretase	Reduce Aβ plaque formation	Wuli et al., 2022
miR-124	Rat pheochromocytoma (PC12) cells; Primary cultured rat hippocampal neurons	BACE1	BACE1/APP	Reduce of Aβ plaque formation	Fang et al., 2012
	Adult male C57BL/6 mice; BV2 mouse microglial cell line; HT22 mouse hippocampal neuronal cell line	RelA	RelA/ApoE signaling pathway	Promote Aβ proteolytic breakdown	Ge et al., 2020
	BV2 microglia cells; SH-SY5Y cells	RFX1	RFX1/ApoE	Improve Aβ clearance	Feng et al., 2017
	Forebrain-specific Dicer conditional knock out mice; Neuro2a cells; Mouse primary cortical neuron	PTBP1	PTBP1/PTBP2/APP	Increase the skip of APP exons 7 and 8 and reduce Aβ plaque formation	Smith et al., 2011
miR-146a	RAW264.7, A549 and HEK293T cells	TLR2	TLR2/A3	Reduce of Aβ levels	Zhang et al., 2015
**Tau protein**					
miR-9a	SH-SY5y neuroblastoma cells; male Tau^P301L-BiFC^ mice	UBE4B	UBE4B/tau	Inhibit tau protein	Subramanian et al., 2021
miR-124	Wild-type neuroblastoma Neuro-2a cells; 6-mon-old male P301S transgenic mice; 2-mon-old male C57BL/6J mice; HEK293/tau cells	PTPN1	miR-124/PTPN1/GSK-3β/PP2A	Increase abnormal phosphorylation of tau protein	Hou et al., 2020
	Transgenic APP/PS1 mice; HCN-2 cell line	CAPN1	miR-124-3p/CAPN1/CDK5	Decrease abnormal phosphorylation of tau protein	Zhou et al., 2019
miR-146a	5xFAD mice	ROCK1	ROCK1/PTEN	Promote tau hyperphosphorylation within neurons	Wang et al., 2016a
**Neuroinflammation**					
miR-9	C57BL/6 N mice	MCPIP1	MCPIP1/NF-κB pathway	Promote microglia activation and exacerbate neuroinflammation	Yao et al., 2014
miR-124	Adult male C57BL/6 mice; mouse BV2 microglial cell line; HT22 mouse hippocampal neuronal cell line	RelA	NF-κB signaling pathway	Promote microglia M2 polarization and reduce neuroinflammation	Ge et al., 2020
	Mouse BV2 microglial cell line	TLR4	TLR4/MyD88/NF-κB p65 signaling pathway	Reduce microglia pro-inflammatory response and reduce neuroinflammation	Yang et al., 2022
	Immortalized human microglia-SV40 cell line	CD14	CD14/TRAM/TRIF/IFN-β	Reduce microglia pro-inflammatory response and reduce neuroinflammation	Pajarskiene et al., 2021
	Hek293 cells; mouse cerebellar granule cells	p38	p38/MAPK signaling pathway	Reduce microglia pro-inflammatory response and reduce neuroinflammation	Pajarskiene et al., 2021
	Primary neural stem cells	DLL4	Notch signaling pathway	Promote astrocyte-to-neuron conversion and reduce neuroinflammation	Jiao et al., 2017
	Primary cortical astrocytes	HES1	SOX9-NFIA-HES1 axis	Promote neuronal differentiation in reactive astrocytes	Zheng et al., 2021b
miR-146a	Human neuronal cells	Complement factor H	Complement factor H-mediated pathway	Lead to an exacerbation of inflammatory processes and reduce neuroinflammation	Lukiw et al., 2008
	Wild-type C57BL/6J and B6C3-Tg (APPswe, PSEN1dE9) male mice	TRAF6	TRAF6/NF-κB	Reduce microglia pro-inflammatory response and reduce neuroinflammation	Mai et al., 2019
**Synaptic dysfunction**					
miR-9	Primary embryonic E18 mouse hippocampal neurons	CAMKK2	CAMKK2-AMPK pathway	Restore Aβ_42_-induced dendritic spine loss	Chang et al., 2014
	Primary embryonic E17 mouse cortical neurons; C57/BL6 and Parkes mice	Map1b	Map1b-mediated pathway	Reduce axon length and promote branching	Dajas-Bailador et al., 2012
miR-29b	Glioblastoma cell line	NAV3	NAV3-mediated pathway	Regulate axon guidance and synapse formation	Jahangard et al., 2020
miR-124	HEK293, BE(2)-M17 and P19 cell lines	ROCK1	ROCK1/PI3K/Akt	Promote the growth of neural protrusions	Gu et al., 2014
	Primary hippocampal neurons	RhoG	RhoG/ELMO/Dock180/Rac1	Axonal and dendritic growth	Franke et al., 2012
	CD63-GFP knock-in mice; primary cultured neurons and astrocytes	miR-132 and miR-218	miR-124-3p suppresses miR-132 and miR-218	Upregulate GLT1 expression and prevent excitotoxicity by clearing excess glutamate around synapses	Men et al., 2019
	Tg2576 transgenic mice	PTPN1	miR-124/PTPN1 interaction	Defect in synaptic transmission and plasticity	Wang et al., 2018
miR-146a	male and female Sprague-Dawley rat pups (P2); C57BL6 male mice	Nlg1	Nlg1-mediated pathway	Inhibit the expression of presynaptic Syt1	Prada et al., 2018
**Neurons**					
miR-9	Aβ25-35-induced AD HT22 cells	GSK-3β	GSK-3β-mediated pathway	Inhibit Aβ_25-35_-induced mitochondrial dysfunction	Liu et al., 2020a
	N1E-115 neuroblastoma cell line	Hairy 1	miR-9/hairy1 pathway	Regulate cell proliferation by modulating the expression of cyclin D and p27 and promote neuronal growth	Bonev et al., 2011
miR-29	A3-treated neuroblastoma cells	TNFAIP1	NF-κB signaling pathway	Attenuate Aβ-induced neuronal death	Liu et al., 2020b
	Glioblastoma cell line (U87); bone marrow- mesenchymal stem cells	BIM	BIM-mediated apoptosis pathway	Exert a regulatory influence on apoptosis and prevent neuron death	Jahangard et al., 2020
	Primary embryonic hippocampal neurons	Brain-derived neurotrophic factor	miR-29c/BDNF	Promote neuronal proliferation	Yang et al., 2015
miR-124	Human neuroblastoma cell lines SK-N-SH and SK-N-BE	BID	miR-124-3p suppresses BID	Reduce of Aβ_25__-__35_-induced apoptosis	Zhang et al., 2021c
	Mouse hippocampal neuronal line (HT22); BV2 mouse microglial cell line CAD cell; Neuro2a cell	ROCK1; ROCK2 PTBP1	ROCK/PTEN/Akt/mTOR PTBP1/PTBP2	Reduce of glutamate-induced neuronal apoptosis Promote neuronal differentiation and nervous system development	Zhu et al., 2024 Makeyev et al., 2007
	Mouse neural stem cells	DACT1	Wnt/3-catenin signaling pathway	Promote proliferation and induces NSC differentiation into neurons	Jiao et al., 2018
miR-146a	Human hippocampal neurons PC12 and cortical neurons	TIGAR STAT1	NF-κB signaling pathway STAT1/MYC pathway	Promote hippocampal neuronal oxidative stress Inhibit total neurite growth and promote apoptosis	Lei et al., 2021 Ma et al., 2021

AMPK: AMP-activated kinase; ApoE: apolipoprotein E; APP: amyloid precursor protein; Aβ: amyloid-β; BACE1: β-site APP-cleaving enzyme-1; BDNF: brain-derived neurotrophic factor; BID: BH3-interacting domain death agonist; BIM: Bcl-2-interacting mediator of cell death; CAMKK2: Ca2+/calmodulin kinase protein 2; CAPN1: Calpainl; CD14: cluster of differentiation 14; CDK5: cyclin-dependent kinase 5; DACT1: dishevelled binding antagonist of beta-catenin 1; DLL4: Delta-like 4; ELMO: engulfment and cell motility; GSK-3β: glycogen synthase kinase-3β; HES1: hairy and enhancer of split 1; IFN-β: interferon-β; Maplb: microtubule-associated protein 1B; MAPK: p38/mitogen-activated protein kinase; MCPIP1: monocyte chemotactic inducing protein 1; mTOR: mechanistic target of rapamycin; MYC: myelocytomatosis oncogene; Myd88: myeloid differentiation primary response gene 88; NAV3: neuron navigator 3; NF-κB: nuclear factor kappa-B; Nlg1: neuroligin1; PP2A: protein phosphatase 2; PS: presenilin protein; PSEN1: presenilin1; PTBP: polypyrimidine tract-binding protein; PTEN: phosphatase and tensin homolog; PTPN1: protein tyrosine phosphatase non-receptor type 1; RelA: the p65 subunit of nuclear factor kappa-B (NF-κB); RFX1: regulatory factor X1; RhoG: Ras homolog family member G; ROCK1: Rho-associated coiled-coil kinase 1; STAT1: signal transducer and activator of transcription 1; TIGAR: TP53-induced glycolysis and apoptosis regulator; TLR: Toll-like receptor; TNFAIP1: tumor necrosis factor-α-inducible protein-1; TRAF6: tumor necrosis factor receptor-associated factor 6; TRAM: Toll-like receptor adaptor molecule; TRIF: Toll-interleukin-1 receptor (TIR)-domain-containing adaptor inducing interferon-3; UBE4B: ubiquitin conjugation E4B.

## Additional files:

***Additional Table 1:*** *Drugs approved by the FDA and representative in the late clinical stage.*

***Additional Table 2:*** *Roles of primary miRNAs in Alzheimer’s disease.*

## Data Availability

*All relevant data are within the paper and its Additional files*.
